# Multivalent Display of Antimicrobial Peptides on Plant Virus Scaffolds Enhances Killing of Drug-Resistant Bacteria

**DOI:** 10.1021/acsnano.6c03117

**Published:** 2026-06-16

**Authors:** Ahmed Ghouneimy, Shahid Chaudhary, Muhammad Tehseen, Ahmed Saleh, Maged Serag, Maya Ayach, Ebtihaj Bukhari, Maazallah Masood, Kosuke Sakashita, Pei-Ying Hong, Satoshi Habuchi, Samir M. Hamdan, Magdy Mahfouz

**Affiliations:** † Laboratory for Genome Engineering and Synthetic Biology, Division of Biomedical Sciences, 127355 King Abdullah University of Science and Technology, 4700 , Thuwal 23955-6900, Saudi Arabia; ‡ Laboratory of DNA Replication and Recombination, Division of Biomedical Sciences, King Abdullah University of Science and Technology (KAUST), Thuwal 23955-6900, Saudi Arabia; § Molecular Imaging and Microscopy Lab, Division of Biomedical Sciences, King Abdullah University of Science and Technology (KAUST), Thuwal 23955-6900, Saudi Arabia; ∥ Imaging & Characterization Core Laboratory, King Abdullah University of Science and Technology (KAUST), Thuwal 23955-6900, Kingdom of Saudi Arabia; ⊥ Analytical Chemistry Core Lab, King Abdullah University of Science and Technology (KAUST), Thuwal 23955-6900, Kingdom of Saudi Arabia; # Water Desalination and Reuse Center, Division of Biological and Environmental Sciences and Engineering, King Abdullah University of Science and Technology (KAUST), Thuwal 23955-6900, Saudi Arabia

**Keywords:** multidrug resistance, antimicrobial peptides, bioproduction, PVX, plant virus, MRSA

## Abstract

Multidrug-resistant (MDR) bacteria pose a significant challenge to global health. Antimicrobial peptides (AMPs) have emerged as promising candidates against MDR bacteria due to their rapid and broad-spectrum activity; however, their clinical translation is hindered by compromised activity, toxicity, and poor stability under *in vivo* conditions. Here, we report the development of RPG (rod-based peptide grids), a plant virus-based antimicrobial platform that harnesses the structural scaffold of high-aspect-ratio *Potato virus X* (PVX) for the multivalent and modular display of AMPs. Our data show that RPG enhances the efficacy of AMPs by more than 9700-fold, maintaining activity under *in vivo* salt conditions. RPG eradicates MDR pathogens within 10–30 min, surpassing the efficacy of last-resort antibiotics (vancomycin, tigecycline, and cefiderocol), while exhibiting low measurable cytotoxicity to mammalian cells at high therapeutic doses. Due to structural complexity, RPG demonstrates stability in serum and resistance to proteases. Multivalent display of peptide variants enabled enhanced broad-spectrum killing at low doses. This work establishes plant virus-AMP conjugates as a safe, potent, broad-spectrum antimicrobial platform, offering a versatile strategy for addressing antibiotic resistance.

## Graphical abstract

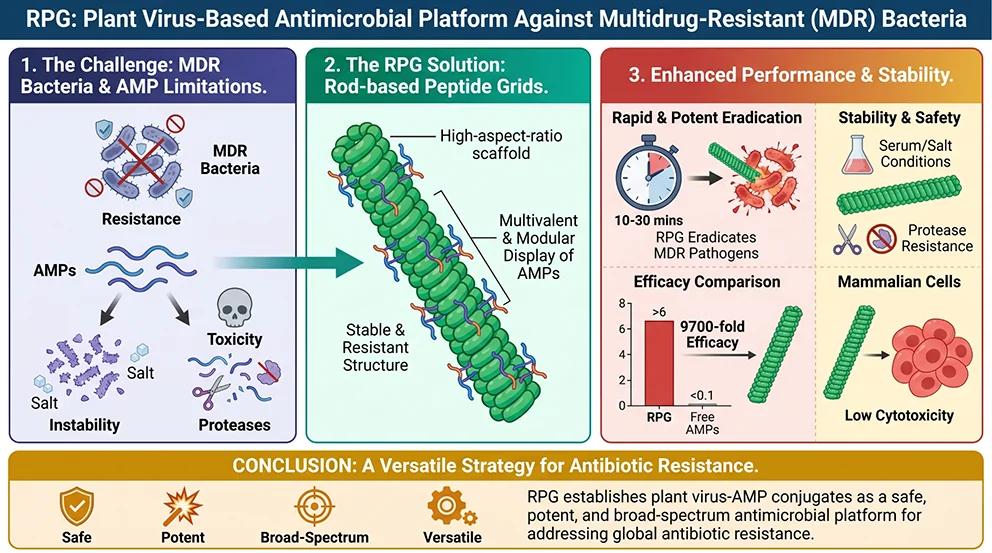

## Introduction

Multidrug-resistant (MDR) pathogens are expected to kill 39 million people globally over the next 25 years.^1^ According to the 2025 reports from the WHO, the fight against MDR is facing a dual crisis: a lack of innovation in drug targets and a scarcity of drug candidates under development.^1–5^ Antimicrobial peptides (AMPs), innate immune effectors found across the tree of life, represent a promising therapeutic class with broad-spectrum activity, rapid bactericidal mechanisms, and low propensity for resistance development.^6,7^ However, the translational potential of AMPs has been hindered by their intrinsic toxicity to mammalian cells, poor proteolytic stability, and reduced activity under *in vivo* conditions, which collectively limit their clinical utility.^8,9^

To overcome these bottlenecks, recent efforts have focused on developing AMPs that are stable through amidation,^10^ cyclization,^11^ noncanonical,^12^ d-amino acid incorporation,^13^ or N-capping modification.^14^ Self-assembly onto nanostructures has increased half-life in serum,^15–17^ while improving the activity of AMPs. Liu et al. assembled cationic peptides around a hydrophobic block of cholesterol and demonstrated a 7-fold potency relative to free peptides.^18^ Meanwhile, peptides assembled around hexadecenoic acid, known as dendrons, showed a 67-fold improvement.^16^

Plant viruses have emerged as a promising class of biocompatible nanocarriers for therapeutic delivery in humans.^19^ Unlike mammalian viruses, plant viruses are inherently noninfectious to humans,^20^ eliminating concerns over pathogenicity or genomic integration.^21^ Their highly ordered, monodisperse structures, exemplified by *Potato virus X* (PVX),^22^ *Cowpea mosaic virus* (CPMV),^23^ and *Tobacco mosaic virus* (TMV),^24^ can be genetically and chemically modified to display peptides, proteins, or drug molecules with high precision. These nanoscale scaffolds exhibit exceptional stability under physiological conditions, favorable biodistribution, and the ability to penetrate tissue barriers, while avoiding the toxicities^25,26^ and immunogenicity often associated with synthetic nanoparticles or bacterial endotoxins. Recent preclinical studies have demonstrated the systemic administration of plant virus nanoparticles in animal models without adverse immune responses,^27^ highlighting their translational potential for applications ranging from oncology to infectious disease and antiviral therapy.^28,29^ Despite these promising attributes, the clinical translation of plant virus-based nanocarriers faces several challenges. First, the immunogenicity of viral coat proteins upon repeated systemic administration remains a practical concern,^30^ though recent work demonstrates that surface engineering strategies, including PEGylation, can effectively attenuate antibody-mediated recognition while preserving therapeutic function.^31,32^ Second, the functionalization efficiency of viral scaffolds is inherently limited by the genetic and steric constraints of coat protein engineering; for example, ribosome-skipping strategies such as F2A yield partial incorporation rates that introduce surface heterogeneity.^33^ Third, large-scale production of plant virus-based platforms raises biosafety considerations around potential environmental release of infectious viral particles.^20^

Here, we develop a modular, plant virus–based AMP platform that addresses both efficacy (activity and stability) and safety barriers. By leveraging PVX as a high-aspect-ratio scaffold, we developed RPG by engineering the PVX coat protein to display SpyCatcher (ePVX) and functionalizing AMPs with SpyTag. We first demonstrate that scaffolding AMPs across ePVX improves their antimicrobial activity compared to free AMPs. After validating the safety profile on mammalian cells including RBCs, we developed multivalent RPG (herein named RPG 2.0) by tagging ePVX with two different AMPs, resulting in a more broad-spectrum platform. We challenge RPG with proteases, serum, salts, and varied storage temperatures, demonstrating robust activity under these conditions. Our work establishes RPG as a versatile antimicrobial platform that combines safety, stability, potency, and broad-spectrum bacterial killing, thereby paving the way for translational applications to address the MDR challenge.

## Results and Discussion

### Engineered PVX Biotemplate for Modular Display Peptides

To engineer a biotemplate for broad-spectrum antimicrobial activity, we applied three key design principles (Figure 1a–b). First, we selected the rod-shaped Potato Virus X (PVX) as a structural scaffold due to its high aspect ratio and safety (noninfectious and nonimmunogenic).^22,25,26^ We fused the SpyCatcher^34^ (domain to the N-terminus of the PVX coat protein, taking advantage of the natural surface exposure of this region).^35,36^ Second, to minimize disruption to viral assembly, we incorporated F2A sequence^33^ upstream of the coat protein. This ribosome-skipping element enables the coexpression of both wild-type and SpyCatcher-fused coat proteins, thereby promoting capsid assembly while still allowing surface functionalization through the incorporation of some coat-SpyCatcher fusions. Third, we inserted a flexible glycine-serine linker^37^ between SpyCatcher and the coat protein to preserve conformational freedom for the displayed antimicrobial peptides (AMPs), thereby facilitating their interaction with bacterial membranes.

**Figure 1:**
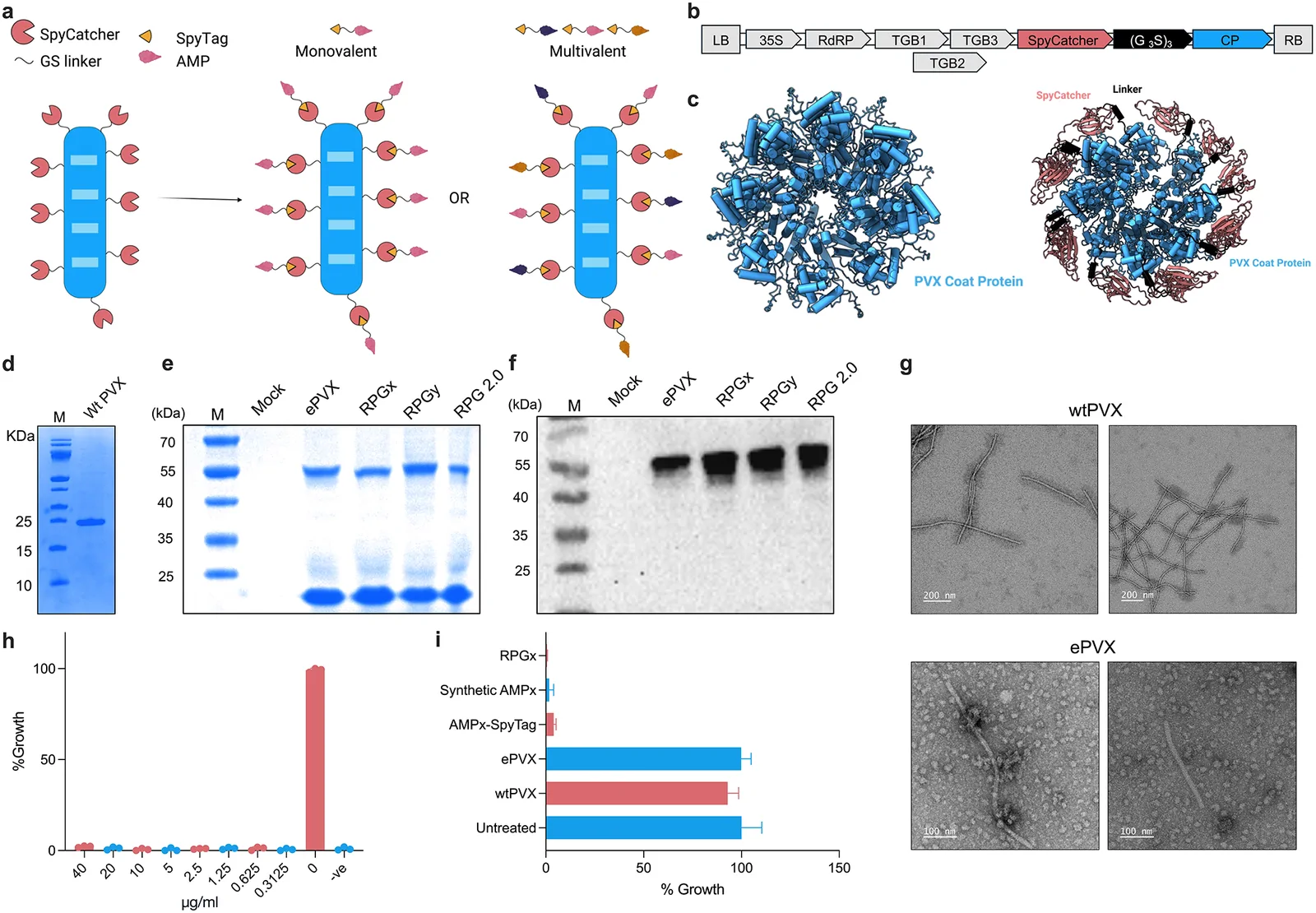
Engineering PVX biotemplate for modular display peptides. a, Schematic illustration of the modular assembly process. The PVX coat protein is genetically fused to SpyCatcher, enabling covalent conjugation with SpyTagged AMPs, either single or multiple peptide variants, on the viral surface. b, Design of the engineered viral parts. SpyCatcher is fused to the N-terminus of the coat protein via an F2A ribosome-skipping sequence and a flexible glycine–serine linker. c, Modeling engineered coat protein with AlphaFold shows that the fusion does not interfere with coat protein multimerization. d–e, SDS–PAGE analysis of PEG-precipitated viral particles showing coat proteins from wtPVX (d) and engineered variants ePVX, RPGx, and RPGy (e). f, Western blot using anti-SpyCatcher antibodies confirms the presence of the SpyCatcher–coat fusion in PEG-precipitated ePVX. g, Transmission electron microscopy (TEM) images showing the characteristic rod-shaped morphology of wtPVX (top; scale bar = 200 nm) and ePVX (bottom; scale bar = 100 nm). h, Antibacterial activity of RPGx against *E. coli* MG1655. i, Testing different parts of RPG on MG1655. All experiments were repeated 3 times independently with *n* = 3. RPG, rod-based peptide grids; ePVX, engineered Potato virus X; wtPVX, wild-type Potato virus X; GS linker, glycine–serine linker.

Structural modeling on Alphafold^38^ predicted that the SpyCatcher fusion does not impact coat protein multimerization, suggesting that engineered PVX (ePVX) would retain its native rod-shaped structure (Figure 1c). To validate this, we expressed wild-type PVX (wtPVX) and ePVX transiently in *Nicotiana benthamiana* via agroinfiltration. Following PEG precipitation^36^ of the virus particles, SDS-PAGE analysis revealed distinct bands corresponding to both wild-type coat (wtCoat) (Figure 1d) and engineered coat (eCoat) (∼50 kDa) (Figure 1e). Interestingly, the calculated molecular weight of eCoat is 40 kDa, while the apparent weight on gel is 50, which we attribute to the presence of putative glycosylation sites.^39^ The relative intensity of the 50 kDa band indicated that 35% of assembled monomers were functionalized with SpyCatcher. Western blotting with anti-SpyCatcher antibodies confirmed the identity of the fusion protein (Figure 1f), and transmission electron microscopy (TEM) revealed properly assembled virions (rod-shaped, uniform structures indistinguishable from wtPVX) (Figure 1g). Collectively, these results confirm the successful production and preserved structural integrity of the ePVX platform.

In a rapid (3 h), room temperature reaction, we conjugated SpyTagged AMPs (Supplementary Note 1 and Figure S1a–c) to ePVX, followed by filtration to remove unbound AMP. Since the size shift is only a 5 kDa difference (50 and 55 kDa), which is below the resolution of SDS-PAGE gels, we sequenced the virus using MS peptide sequencing and confirmed proper fusion (Figure S1d–e). The resulting AMP-decorated ePVX is hereafter named RPG (rod-based peptide grid).

Having established structural integrity, we then sought to assess whether RPG is effective as an antibacterial. We cocultured varying concentrations of RPGx (ePVX tagged with AMPx) with an initial inoculum of *E. coli* MG1655. Remarkably, RPG eradicated 100% of *E. coli* at concentrations as low as 0.312 μg/mL (Figure 1h). In contrast, wtPVX or ePVX did not exhibit measurable killing (Figure 1i). This indicates that the observed killing is attributed to the functional elements on the surface of ePVX. Together, these results establish RPG as a structurally intact, modular PVX-based platform capable of displaying functional AMPs with antibacterial activity.

### RPG Demonstrated Robust Killing of MDR Pathogens

Having established RPG’s structural and functional activity, we evaluated the antimicrobial activity of RPGx (conjugated to AMPx—a peptide with membranolytic, antibiofilm, and immunomodulatory function^10,40,41^ ), RPGy (conjugated to AMPy—a peptide with immunomodulatory function^42^), and RPGz (conjugated to AMPz—a peptide derived from AMPx^43^). These constructs were assessed alongside their corresponding synthetic peptides (AMPx, AMPy, and AMPz) against a panel of MDR pathogens (*E. coli* *PI7*,^44^ methicillin-resistant *S. aureus* (MRSA) USA300, *K. pneumoniae*, *A. junii*, *P. aeruginosa*, and *E. faecalis*). We performed a standard bacterial killing assay to assess their efficacy.^45^ Briefly, we cocultured each of the MDR pathogens with RPGx at varying concentrations for 24 h. RPGx, RPGy, and RPGz successfully inhibited the growth of MDR pathogens at concentrations ranging from <1.5 μg/mL to 100 μg/mL (Figure 2a; S2; S3; S4).

**Figure 2:**
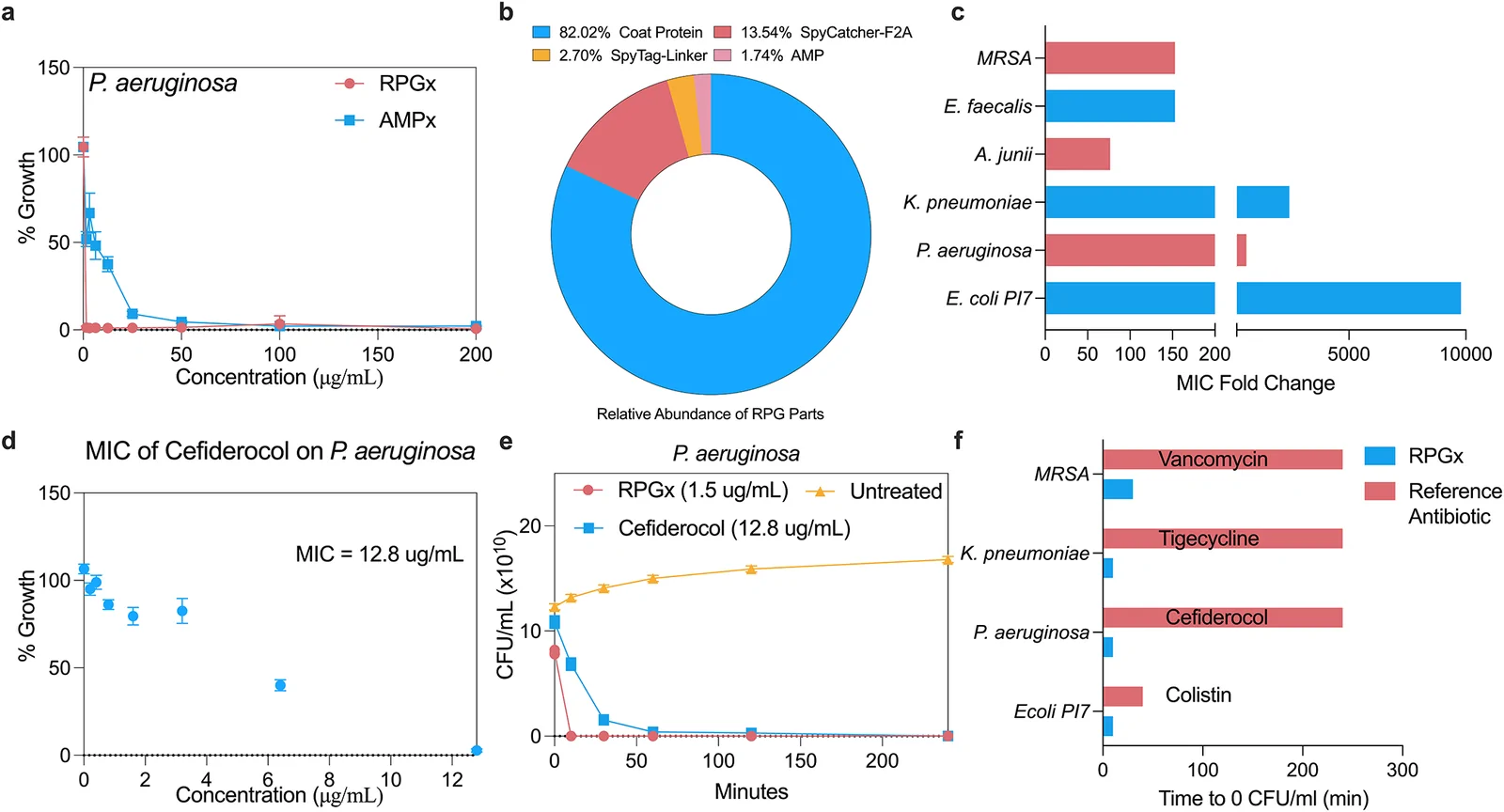
Antimicrobial activity of RPG. a, Minimum inhibitory concentration (MIC) of RPGx and free AMPx against *P. aeruginosa*. b, Relative abundance of RPG components estimated from semiquantitative SDS–PAGE band quantification, assuming ∼100% AMP conjugation efficiency. c, Fold change of MIC of conjugated AMPx relative to free AMPs. Fold change was calculated by dividing the MIC of free AMPx by the MIC of the actual concentration conjugated AMPx (AMPx present on the viral surface). d, MIC of cefiderocol on *P. aeruginosa*. e, Kinetic time points of eradication of *P. aeruginosa* using RPGx and cefiderocol at MIC. f, Kinetics end points of eradicating an initial inoculum of 10^11^ CFU/ml of MDR pathogens using RPGx vs the reference antibiotic. All experiments are repeated three times independently with *n* = 3. RPG, Rod-based Peptide Grids; MIC, minimum inhibitory concentration; MDR, multidrug-resistant; CFU, colony-forming unit.

Because AMPs, the functional part of RPG, represent around 3% of the virus conjugate (Figure 2b), the effective dose of AMPs is less than that of the nominal MIC value of RPG. For example, RPGx kills *E. coli* *PI7* at an MIC of <1.5 μg/mL (Figure S2), corresponding to 0.047 μg/mL of AMPx (Figure S2). Compared to the MIC of free AMPx (200 μg/mL) (Figure S2), RPG utilizes >9700-fold less AMPs to achieve similar levels of killing (Figure 2c). Across all tested MDR pathogens, the effective MIC of conjugated AMPs was 153–9792-fold lower than that of their free counterparts (Figure 2c, S2h). We argue that the high local density of positive charges on the virus enhances electrostatic interactions with bacterial membranes, thereby resulting in reduced MIC values and stronger antimicrobial activity.^46–48^

We then sought to investigate the killing kinetics of RPGx to benchmark against well-established clinical antibiotics. We exposed each MDR pathogen to RPGx or its corresponding last-resort antibiotic: vancomycin for MRSA, tigecycline for *K. pneumoniae*, and cefiderocol for *P. aeruginosa*. RPGx eradicated the initial inoculum of 10^11^ in less than 10–30 min, whereas conventional antibiotics required ≥ 240 min to achieve comparable killing under identical conditions (Figure 2d–f; S5; Table S1).

Altogether, these results establish RPG as a highly potent and fast-acting antimicrobial platform that outperforms both free AMPs and last-resort antibiotics. We’ve tested the RPG platform on peptides that assume α-helical and/or β-sheet structures. Testing other classes of AMPs with different structures (circular, linearized, mixed α-helical and β-sheet conformations) would reveal the generalizability of the RPG platform.

### RPG Exhibited Low Cytotoxicity toward Mammalian Cells

AMPs pose an inherent risk of off-target toxicity due to their membrane-disrupting activity in mammalian cells.^49^ This necessitates rigorous biocompatibility testing before advancing to clinical applications. To assess cytotoxicity, we performed a standard hemolysis assay where human red blood cells were treated with increasing concentrations of RPGx, RPGy, or free AMPx/AMPy controls (Figure 3a; Figure S6a). All constructs showed dose-dependent hemolysis, but critically, hemolysis remained negligible at MIC values (Figure S2h)—demonstrating a safe therapeutic range. IC_50_ values (concentration causing 50% hemolysis) were: RPGx = 258.6 μg/mL, RPGy = 280.1 μg/mL, compared to free AMPx = 326.5 μg/mL and free AMPy = 372.5 μg/mL. It is important to note that conjugation of AMPs to the PVX scaffold (RPG) results in a measurable increase in cytotoxicity relative to free peptides. Semiquantitative analysis indicates that ∼90-fold lower concentrations of conjugated AMPx are sufficient to induce 50% hemolysis in RBCs compared to their free counterparts (Figure S6). Despite this increase in intrinsic cytotoxicity, the IC_50_ values remain substantially higher than the corresponding MICs across all tested pathogens (Figure S2h and S3a–f), supporting a favorable therapeutic index.

**Figure 3:**
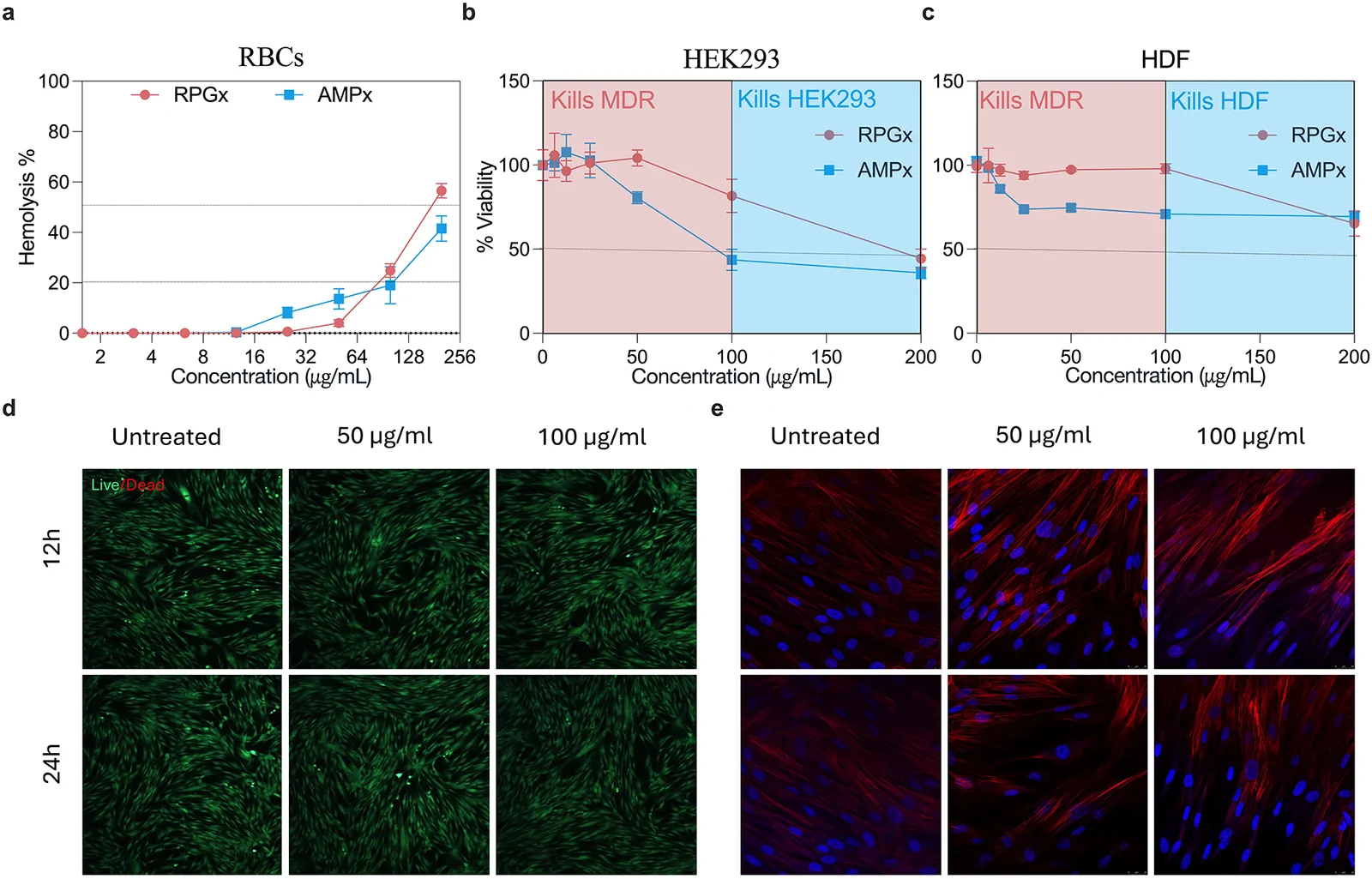
Cytotoxicity of RPGx and AMPx on mammalian cell lines. a, Hemolytic activity of different concentrations of RPGx or AMPx on red blood cells (RBCs). b-c, Cell viability assay testing different concentrations of RPGx or AMPx on HEK293 (b**)** or HDF (c) cell lines. d, Live/dead staining of HDF cells treated with RPGx. e, Cytoskeleton staining of HDF cells treated with RPGx and visualized with a confocal microscope. All experiments are repeated three times independently with *n* = 3. RPG, rod-based peptide grids; HDF, human dermal fibroblasts; HEK293, human embryonic kidney cells; RBCs, red blood cells.

To further assess cytotoxicity, we cocultured RPGx or AMPx with three cell lines: HEK293 (adherent), Fibroblast (adherent), and K562 (suspension) cells (Figure 3b–c; S6b). At a concentration of 50 μg/mL, no adverse effects were detected, as indicated by Presto Blue cell viability assay. A slight decrease in viability was observed at concentrations equal to or above 100 μg/mL. The estimated IC50 of RPGx is 200 μg/mL in HEK293 and K562 cells, and more than 200 μg/mL in HDF cells. Compared to AMPx, RPGx was either safer (in HEK293 and HDF cells) or showed a similar safety profile (in K562 cells).

To further validate the safety of RPGx, we sought to visualize the cells to detect any morphological changes. HDF cells treated with 50 and 100 μg/mL of RPGx were similar to the untreated control in live (green)/dead (red) staining (Figure 3d). While viability and live/dead staining reveal that RPGx was safe at concentrations below 200 μg/mL for HDF cells, we hypothesized RPGx could be affecting the morphology of the cells. To this end, we cocultured RPGx with HDF cells and then stained the cells with rhodamine-phalloidin, which stains F-actin. Confocal microscopy images revealed no alterations in cell morphology, reinforcing the low cytotoxicity of RPGx on mammalian cells (Figure 3e). Collectively, our data suggest that RPGx is safe for mammalian cells at concentrations below 100 μg/mL.

We attribute the selective activity of RPG against bacterial cells to differences in surface charge between prokaryotic and eukaryotic membranes. Bacterial cytoplasmic membranes are enriched in anionic phospholipids, such as phosphatidylglycerol and cardiolipin, resulting in a net negative surface charge that promotes the electrostatic attraction of cationic RPG. In contrast, the outer leaflets of mammalian cell membranes are dominated by zwitterionic phospholipids, including phosphatidylcholine and sphingomyelin, which reduce nonspecific peptide binding.^50,51^

### RPG 2.0: Multivalent Tagging of AMPx and AMPy on ePVX Results in Broad-Spectrum Killing

Because RPGx and RPGy have different killing efficiencies against different MDR pathogens, we sought to display both AMPx and AMPy simultaneously. We observed that RPGx is more effective at killing *E. coli* *PI7*, while RPGy is more efficient at killing MRSA and *E. faecalis* (Figure S2, S3). We hypothesized that tagging ePVX with both AMPx and AMPy would improve the activity of both peptides, resulting in a more effective broad-spectrum activity. To this end, we have tagged equimolar amounts of AMPx and AMPy on ePVX and proceeded with filter purification to remove excess unbound AMPs (Figure S7). We named the resulting multiplexed conjugate as RPG 2.0. In a head-to-head comparison, we cocultured MDR pathogens with either RPG 2.0 or a mixture of AMPx and AMPy. We have observed potent killing at lower concentrations with RPG 2.0, resulting in a 306–4759-fold reduction of the MIC of conjugated peptides (Figure 4a–b; S8; Table S2; Table S3; Table S4 MIC comparison tables).

**Figure 4:**
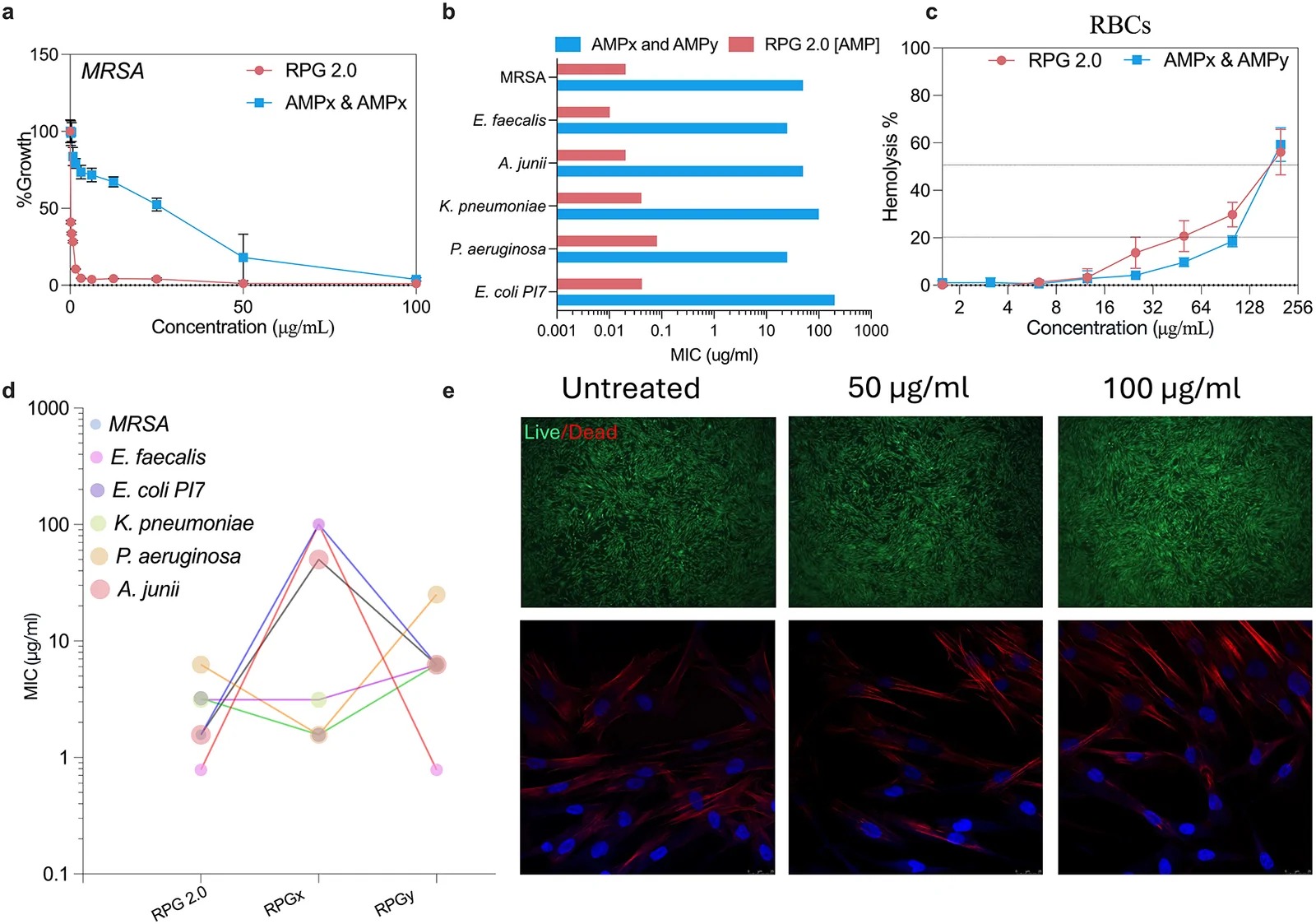
Functional characterization of RPG 2.0. a, MIC of RPG 2.0 vs mixed AMPx and AMPy on MRSA. b, MIC of conjugated AMPs on RPG 2.0. relative to free AMPx and AMPy. c, Assessing the hemolytic activity of RPG 2.0 vs free AMPx and AMPy using a standard hemolysis assay on RBCs. d, Comparing the MIC value of RPGx, RPGy, and RPG 2.0 on 6 MDR pathogens. e, Live/dead staining of HDF cells treated with RPG 2.0 (top). Cytoskeleton staining of HDF cells treated with RPG 2.0 and visualized with a confocal microscope (bottom). All experiments were repeated three times independently with *n* = 3. RPG, rod-based peptide grids; MIC, minimum inhibitory concentration; MRSA, methicillin-resistant *Staphylococcus aureus*; RBCs, red blood cells.

Multivalent RPG 2.0 indeed improved the killing activity of both RPGx and RPGy. For instance, RPGx kills MRSA at MIC of 100 μg/mL, in contrast to RPGy’s MIC of 6.25 μg/mL. RPG 2.0 enhanced both activities, resulting in a significantly reduced MIC of 1.56 μg/mL. Overall, RPG 2.0 eradicated MDR pathogens tested at concentrations below 6.25 μg/mL (Figure 4d).

We then sought to assess the cytotoxicity of RPG 2.0 on mammalian cells. We evaluated cytotoxicity using a conventional hemolysis assay in which human erythrocytes were exposed to increasing concentrations of RPG 2.0 or free peptide controls (AMPx & AMPy). All formulations induced hemolysis in a concentration-dependent manner (Figure 4c); however, lytic activity was minimal at concentrations equivalent to the MIC (Figure 4a–b), indicating a favorable therapeutic window. The half-maximal hemolytic concentrations (IC_50_) were determined as follows: RPG 2.0, 189.1 μg/mL; free AMPx & AMPy, 248.2 μg/mL. Further coculturing varying concentrations of RPG 2.0 (1.56–200 μg/mL) with HDF cells showed a slight decrease in viability after 100 μg/mL, 16-fold higher than the largest MIC (6.25 μg/mL on *P. aeruginosa*) (Figure S8h). It is imperative to note that conjugated AMPs (RPG) exhibit a measurable increase in cytotoxicity relative to free AMPs. Semiquantitative comparison of conjugated AMPs vs free AMPs reveals that 90 times less conjugated AMPs are needed to kill 50% of RBCs, while 70-fold less are needed to kill HDF cells (Figure S9). Nonetheless, the IC50 values remain substantially higher than the MIC across all tested pathogens (Figure S9; Figure 4b), supporting a favorable therapeutic index.

To further validate the cytotoxicity profile of RPG 2.0, we examined its effects with live/dead and cytoskeleton staining. HDFs treated with 50 or 100 μg/mL of RPG 2.0 exhibited staining patterns in live (green)/dead (red) assays comparable to untreated controls (Figure 4e). To investigate potential morphological changes, we cocultured HDFs with RPG 2.0 and stained with rhodamine–phalloidin to visualize F-actin structures. Confocal microscopy revealed no detectable alterations in cytoskeletal organization or cell morphology, further confirming the minimal cytotoxicity of RPG 2.0 in mammalian cells (Figure 4e). Together, these findings indicate that RPG 2.0 is well tolerated by HDF cells at concentrations up to 100 μg/mL. Altogether, these data suggest that multiplex tagging of different AMPs could 1) have effective broad-spectrum killing, 2) exhibit low MIC values, and 3) maintain a good safety profile in mammalian cells.

### RPG 2.0 Permeabilized Bacterial Cell Wall

Having established RPG 2.0 as a safe, effective, broad-spectrum platform, we sought to investigate the mechanism of bacterial killing. Synthetic AMPx, the peptide displayed multivalently on the PVX scaffold, kills bacteria through a classical carpet-like mechanism: initial electrostatic attraction to the anionic bacterial surface, followed by insertion into and disruption of the cytoplasmic membrane, culminating in rapid loss of membrane.^10^ Because scaffolding across PVX could theoretically alter AMP interaction geometry, we investigated whether RPG 2.0 retains this membrane disruption mode of action.

We first assessed membrane permeabilization using propidium iodide (PI), a DNA-intercalating dye that cannot traverse intact membranes. We incubated RPG 2.0-treated MRSA with propidium iodide and performed flow cytometry to measure the uptake of the PI dye (Figure 5a–b). RPG 2.0 induced >70% PI incorporation, confirming membrane disruption. This activity is comparable to that of ethanol-treated bacteria, which we used as a positive control for complete membrane compromise (Figure 5b; S10). By contrast, vancomycin, a glycopeptide antibiotic that inhibits cell wall biosynthesis without directly perturbing the cytoplasmic membrane,^52^ accumulated 35.2% PI, a value close to that of untreated MRSA. The difference in PI dye accumulation suggests increased membrane permeability and potential disruption to the membrane integrity.

**Figure 5:**
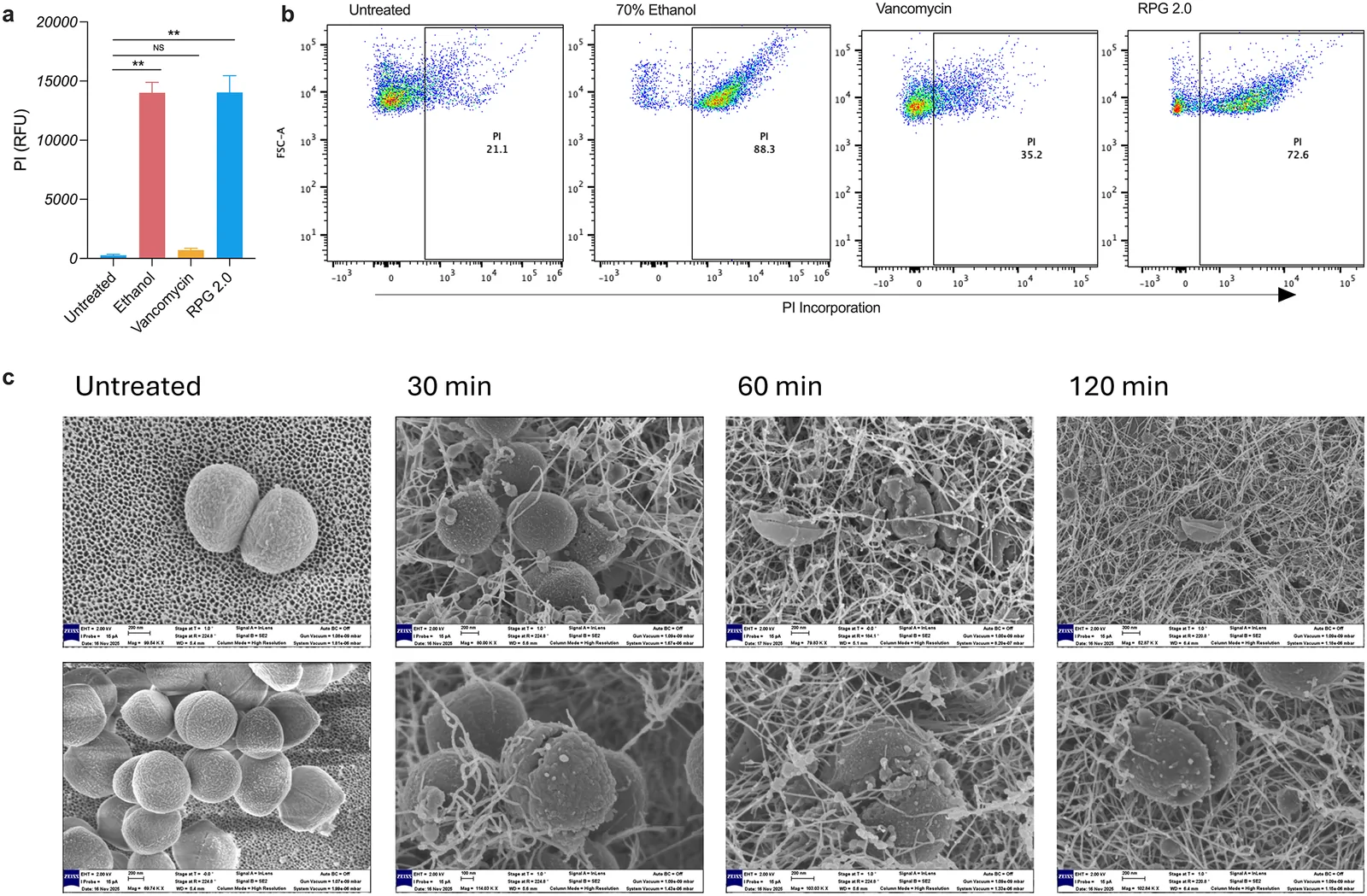
RPG 2.0 permeabilized bacterial cell wall. a, MRSA (10^11^ CFU/ml) was treated with 70% ethanol, vancomycin, or RPG 2.0 at MIC and incubated for 2 h. PI was then added and incubated for 30 min in the dark, and quantified with a spectrophotometer. Asterisks represent a *P* value <0.05. b, MRSA (10^11^ CFU/ml) was treated with 70% ethanol, vancomycin, or RPG 2.0 at MIC and incubated for 2 h. PI was then added and incubated for 30 min in the dark, and samples were analyzed with a flow cytometer. RPG, rod-based peptide grids; MRSA, methicillin-resistant *Staphylococcus aureus*; PI, propidium iodide; MIC, minimum inhibitory concentration.

We further assessed outer membrane permeabilization using the 1-*N*-phenylnaphthylamine (NPN) fluorescence probe. NPN penetrates selectively into the hydrophobic interior of compromised membranes, producing an increase in fluorescence intensity.^53^ RPG 2.0-treated MRSA USA300 showed a marked increase in NPN fluorescence compared to untreated cells (Figure S11), confirming that RPG 2.0 disrupts the outer membrane. In contrast, vancomycin-treated cells, where killing proceeds through inhibition of cell wall biosynthesis rather than direct membrane perturbation,^52^ showed NPN accumulation comparable to untreated controls.

To validate these findings, we used scanning electron microscopy (SEM) to visualize bacterial cells before and after treatment with RPG 2.0. Untreated cells displayed the expected smooth, turgid morphology with intact spherical envelopes characteristic of healthy *staphylococci*. In contrast, following RPG 2.0 treatment, cell membranes exhibited visible surface damage, loss of curvature, and overall cell shrinkage (Figure 5c–e). Many cells appeared deflated or shrunken, indicating osmotic imbalance and leakage of cytoplasmic contents. These results suggest that RPG 2.0 functions by targeting the bacterial membrane, inducing permeabilization, and ultimately causing cell death.

Although our data clearly show membrane disruption starting at 30–60 min of incubating RPG with bacteria, it would be reductive to attribute RPG’s antibacterial activity solely to membrane perturbation. In our kinetic assays, RPG eliminated MRSA within 30 min—at which point there are still some visibly intact bacterial cells (Figure S5b). This suggests that additional nonmembranolytic processes may be operating in parallel to accelerate bacterial clearance. A growing body of evidence has shown that AMPs can interfere with multiple essential functions of the bacterial envelope, including inhibiting cell wall biosynthesis, cell division, and lipopolysaccharide transport trafficking.^54^ Further investigations into the mechanism of action of RPG could identify a novel method of bacterial targeting, which is needed to predict resistance pathways.

To directly test whether the antimicrobial activity of RPG is dependent on surface charge density, we preincubated RPG 2.0 with heparin (a highly sulfated polyanion) at increasing concentrations. Heparin directly neutralizes cationic surface charge through electrostatic binding. Heparin treatment resulted in a concentration-dependent reduction of RPG 2.0 antimicrobial activity, with complete activity loss at high heparin concentrations (Figure S12).

### RPG 2.0 Is Stable in Serum, Resists Proteases, Maintains Activity under Physiologic Salt Conditions, and Exhibits Long-Term Stability

AMPs suffer from susceptibility to protease cleavage, reduced activity under *in vivo* conditions, and instability in serum.^55–57^ We reasoned that scaffolding AMPs on the ePVX scaffold could shield them from proteases, endow them with stability in serum, and preserve their activity under physiological salt conditions. First, we hypothesized that the complex structure of RPG2.0 would be resistant to protease degradation, as proteases tend to work on linear structures.^58,59^ Indeed, we incubated RPG 2.0 with chymotrypsin (1.25–2.5 mg/mL) for 2 h and then cocultured the solution with MRSA. We observed minor changes to the MIC value, increasing from 3.125 to 6.25 μg/mL in the highest concentration of chymotrypsin, indicating stability under these conditions (Figure 6a).

**Figure 6:**
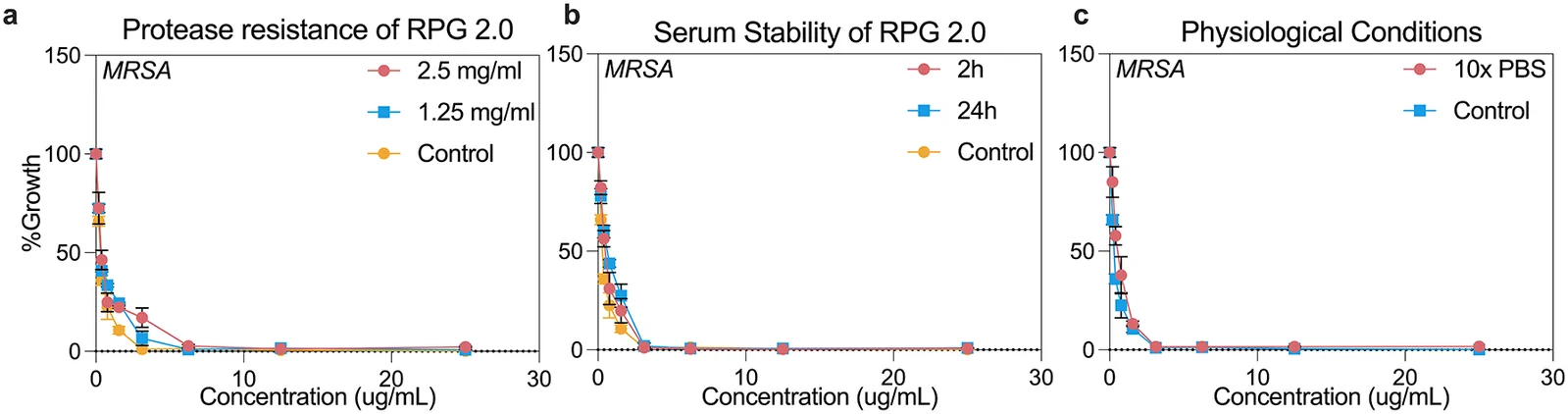
Stability of RPG 2.0. a, RPG 2.0 was exposed to varying concentrations of chymotrypsin and incubated for 2 h, followed by MIC assay on MRSA. b, RPG 2.0 was exposed to 100% serum for 2 or 24 h, followed by MIC assay on MRSA. c, MIC assay was conducted in MHB media supplemented with 10x PBS to mimic physiologic conditions. All experiments were repeated three times independently with *n* = 3. RPG, rod-based peptide grids; MIC, minimum inhibitory concentration; MRSA, methicillin-resistant *Staphylococcus aureus*.

Subsequently, we investigated the stability of RPG 2.0 in human serum. Serum contains a high concentration of anionic proteins and diverse proteases that readily bind to or degrade AMPs, resulting in half-lives typically ranging from only a few minutes to several hours.^60^ To this end, we incubated equal volumes of RPG 2.0 and 100% serum for 2 and 24 h. Coculturing the solution with MRSA revealed no significant change in the MIC value, indicating exceptional stability of RPG 2.0 in serum (Figure 6b).

The antibacterial activity of AMPs is highly dependent on salt composition, and many peptides lose their efficacy under physiological conditions, mainly because of high ionic strength and the presence of divalent cations or polyanions.^54–57^ Early studies demonstrated that the microbicidal activity of host-defense peptides such as the human cathelicidin LL-37 and β-defensins is substantially impaired at physiological salt concentrations, particularly in the presence of divalent cations.^61,62^ To evaluate the salt effect, we supplemented MHB media with 10× PBS to mimic physiologic salt conditions and then conducted standard killing assays. We observed no change in the MIC value, indicating that scaffolding AMPs on PVX overcame the charge-shielding effect of physiologic salts (Figure 6c). The reason could be attributed to the high charge density on the surface of PVX, which in turn could enhance the adsorption between RPG 2.0 and negatively charged microbial membranes.^55^

Although RPG demonstrates robust activity under *in vivo* simulated conditions, its therapeutic potential requires further exploration in murine models of infection. Despite being noninfectious in humans, the multivalent, repetitive PVX coat protein displays pathogen-associated molecular patterns (PAMPs) that are recognized by the immune system, triggering innate immune responses, particularly upon repeated administration.^30^ However, recent work has shown that surface engineering effectively attenuates antibody-mediated immune recognition while preserving therapeutic function. Shi et al. demonstrated that polydopamine coating of TMV nanoparticles reduces recognition by both preexisting and newly generated anti-TMV antibodies while maintaining full MRI and optical imaging capability across multiple doses.^31^ We leverage this precedent to propose that, for the RPG platform, the displayed cationic AMPs themselves may serve as an immune-evasion mechanism; the amphipathic nature and charge clustering of these peptides may alter epitope accessibility and immune cell recognition patterns. Beyond peptide-mediated evasion, additional surface modification strategies such as PEGylation^32^ can be applied to avoid premature clearance.

Long-term storage stability of RPG formulations is important for practical application and development. Recent work by the Steinmetz group demonstrates that plant virus nanoparticles are stable up to 2 years.^63^ We have systematically evaluated RPG storage stability at 4 °C, −20 °C, and in lyophilized format (Figure S13). RPG maintained full antimicrobial activity when stored at 4 °C over the 3-week testing period. Lyophilization with 2% trehalose followed by 4 °C storage resulted in modest of function, with MIC values tripling by day 21. Storage at −20 °C caused partial loss of functionality by the end of the testing period. These results established 4 °C storage as the optimal near-term approach. However, the modest potency loss upon lyophilization and freezing suggests that formulation optimization can recover this stability.

### Biophysical Characterization of RPG Reveals Local Clustering of AMP Conjugations

The antimicrobial activity of cationic AMPs is fundamentally driven by electrostatic interactions with negatively charged bacterial membranes,^6,54,64,65^ where surface charge density directly correlates with bactericidal potency. To characterize the structural and surface properties of our modality, we employed zeta potential measurements and atomic force microscopy (AFM). Zeta potential analysis quantifies the surface charge evolution from wtPVX to ePVX to RPG, providing direct evidence for the cationic charge enhancement that underlies the observed fold improvements in antimicrobial activity. In addition, AFM offers nanoscale-resolution topographical characterization, validating the preservation of rod-shaped morphology while revealing unique surface modifications associated with SpyCatcher functionalization and AMP conjugation. Together, these biophysical techniques establish the structure–function relationship between surface charge density, structural integrity, and antimicrobial efficacy.

To address this directly, we repeated the zeta potential measurements under low ionic strength conditions (0.0005 M KP buffer) to minimize counterion shielding and reveal the intrinsic surface charge. Under these conditions, a clear positive charge trend emerged: wtPVX (mean = +0.47 mV), ePVX (mean = +28.6 mV), and RPGx (mean = +40.8 mV). These results confirm that the progressive functionalization from wtPVX to ePVX to RPGx does indeed confer increasingly positive surface charge, which was masked in the original measurements by the high ionic strength of 0.1 M KP buffer.

Zeta potential measurements revealed an increase in surface charge from wtPVX to ePVX to RPGx (Figure 7a). Wild-type PVX exhibited a mean zeta potential of +0.47 mV. Genetic modification to incorporate SpyCatcher resulted in an increase in positive charge, with ePVX displaying a zeta potential of +28.6 mV. Subsequent conjugation with cationic AMPx further increased the surface positive charge, yielding RPGx with a zeta potential of +40.8 mV. The stepwise increase in positive surface charge correlates with the engineered modifications. The 28.13 mV increase upon SpyCatcher fusion (wtPVX to ePVX) reflects the introduction of positively charged residues within the SpyCatcher domain. The additional 12.2 mV increase following AMP conjugation (ePVX to RPGx) confirms successful surface decoration with cationic antimicrobial peptides (Supplementary Note 2).

**Figure 7:**
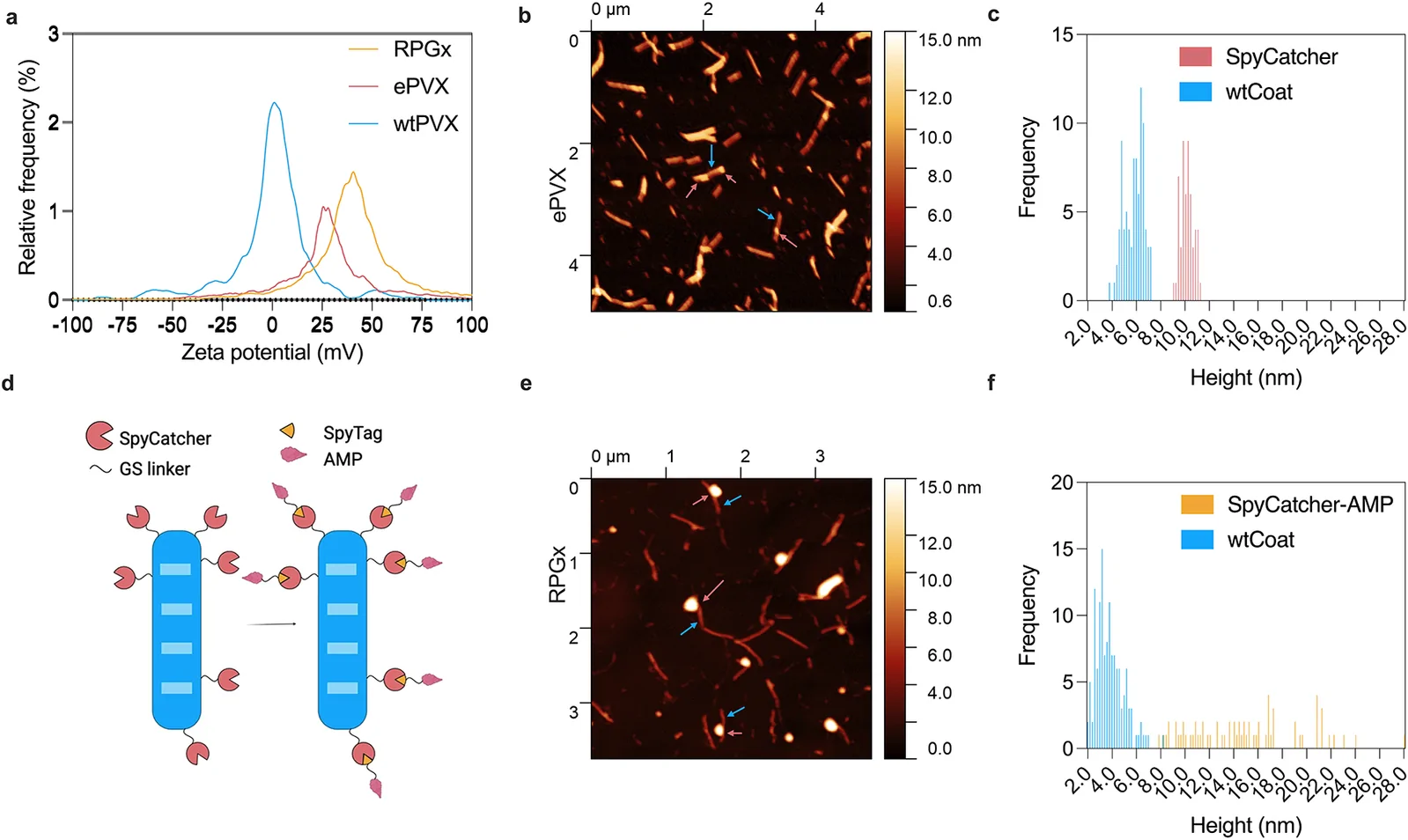
Biophysical characterization of RPG. a, Frequency distribution of zeta potential measurements for wtPVX (mean = +0.47 mV), ePVX (mean = +28.6 mV), and RPGx (mean = +40.8 mV) in 0.5 mM KP buffer. b, AFM characterization showing multiple intact rod-shaped ePVX particles. Blue arrows indicate wild-type virion regions, whereas red arrows indicate visible SpyCatcher decoration. c, Height distribution of ePVX regions without visible SpyCatcher decoration (mean = 5.83 ± 0.81 nm) and regions with SpyCatcher protrusions (mean = 10.10 ± 0.53 nm). d, Schematic representation of PVX-AMP conjugations after AFM analysis showing a heterogeneous distribution of SpyCatcher units. e, AFM image showing RPGx particles with visible decoration. Blue arrows indicate virions, whereas red arrows indicate SpyCatcher-SpyTag-AMP. f, Height distributions for decorated (mean = 14.95 ± 4.53 nm) and bare (mean = 3.84 ± 1.16 nm) regions of RPGx. RPG, Rod-based Peptide Grids; wtPVX, wild-type Potato virus X; wtCoat, wild-type coat; ePVX, engineered Potato virus X; AFM, atomic force microscopy.

To characterize the morphology and structural integrity of ePVX, we performed dry-phase atomic force microscopy (AFM) imaging. ePVX particles retained their rod-shaped morphology (Figure 7b). AFM also measures the height (diameter), which is a good indicator of conjugation events. Notably, individual particles displayed heterogeneous surface features, with some regions exhibiting visible protrusions consistent with SpyCatcher decoration. Height analysis revealed two distinct populations: regions without apparent SpyCatcher decoration measured 5.83 ± 0.81 nm (Figure 7c) while regions with visible SpyCatcher protrusions measured 10.10 ± 0.53 nm (Figure 7b–c). The height difference of approximately 4.3 nm between decorated and SpyCatcher-free regions of ePVX is consistent with the addition of SpyCatcher domains (∼15 kDa, expected diameter 3–4 nm). The observation of heterogeneous SpyCatcher distribution across ePVX particles is consistent with the F2A ribosomal skipping strategy employed in this study. The F2A sequence enables stochastic coexpression of wild-type and SpyCatcher-fused coat proteins, resulting in mosaic particles with variable SpyCatcher density.

RPGx particles maintained their rod-shaped morphology, with visible surface features consistent with AMP conjugation (Figure 7e). Height measurements of decorated regions yielded 14.95 ± 4.53 nm, representing an increase of approximately 5 nm compared to ePVX-decorated regions, consistent with the addition of SpyTag-AMPs (Figure 7f).

AFM imaging provided direct visualization of the progressive structural modifications from ePVX to RPGx. The preservation of rod-shaped morphology across the two constructs confirms that SpyCatcher fusion and AMP conjugation do not prevent viral assembly or disrupt capsid architecture. The height increases from ePVX-decorated regions (10.10 nm) to RPGx-decorated regions (14.95 nm) represents an increase of approximately 5 nm, suggesting successful AMP conjugation. This height increase, which exceeds the expected size of individual SpyTag-AMP molecules (2–3 nm for a 5 kDa peptide), combined with the large standard deviation (±4.53 nm), indicates that AMPs may cluster or stack on the viral surface (where there happen to be adjacent SpyCatcher units) rather than forming a uniform monolayer (Supplementary Note 3).

The high efficiency of RPG might be attributed to the high local concentration of antimicrobial peptides on the surface of ePVX. Cationic AMPs disrupt bacterial membranes primarily through electrostatic interactions with anionic surface components, such as lipopolysaccharides, in Gram-negative bacteria.^46^ These interactions destabilize the lipid bilayer, compromising membrane integrity and ultimately leading to the leakage of cellular content.^46^ Several mechanistic models have been proposed to describe what happens on the cell surface, including the carpet, aggregation, toroidal pore, and barrel-stave models,^47^ which collectively explain how AMPs permeabilize bacterial membranes once a critical surface concentration is reached, directly correlating with their antimicrobial potency.^48^ In the case of RPG, the high-aspect-ratio virus scaffold delivers a high dose of AMPs at any point where RPG exists, making it more efficient at achieving the critical concentration of AMPs required for membrane disruption. AFM analysis further revealed that AMPs cluster on the surface of ePVX, rather than forming a uniform layer. Particles with clustered AMPs may deliver even higher local charge that is more effective than particles with uniformly sparse coverage.

The capacity to deliver and create high local concentrations of AMPs improved their efficacy and maintained potency even in the presence of physiological salts. Previous studies have scaffolded antimicrobial peptides around nanostructures, demonstrating potent killing and stability in serum. Liu et al. clustered cationic peptides around a hydrophobic block of cholesterol and demonstrated 7-fold potency relative to free peptides.^18^ Meanwhile, peptides clustered around hexadecenoic acid, known as dendrons, showed a 67-fold improvement, with significantly reduced activity under *in vivo* salt conditions.^16^ Peptide-conjugated silver nanoparticles were also highly potent antimicrobials. However, metal nanoparticles, while effective in various applications, pose disadvantages such as potential toxicity to human cells, production challenges, and limited biodegradability in the human system, which can hinder their long-term therapeutic use.^66,67^ RPG utilizes rod-shaped, high-aspect-ratio PVX, which theoretically provides 1270 coat protein subunits amenable to conjugation.^68–71^ The result is a potent, biocompatible antimicrobial agent with more than 9700-fold increased potency toward MDR species.

The F2A ribosome-skipping strategy employed in this study results in approximately 35% incorporation of SpyCatcher-fused coat proteins, producing mosaic particles with heterogeneous AMP surface density.^33^ While this partial incorporation is sufficient to drive the up to 9700-fold improvements in antimicrobial potency, high.er incorporation rates could theoretically further increase local AMP density and enhance bactericidal activity. Importantly, the stochastic nature of F2A-mediated skipping may lead to batch-to-batch variation. Direct fusion strategies could theoretically yield 100% decoration with less variation, potentially resulting in more potent antimicrobial drug candidates.

## Conclusion

Here, we show that scaffolding antimicrobial peptides across high-aspect-ratio plant viruses yields potent, yet safe, broad-spectrum antimicrobial agents that eradicate MDR pathogens at low concentrations. Rather than antimicrobial peptide engineering, we offer a strategy for peptide scaffolding that unlocks latent antimicrobial potential. The clinical translation of AMPs has been constrained by diminished activity under physiological salt conditions, susceptibility to proteolytic degradation, and off-target toxicity at therapeutic concentrations. Our scaffold, named RPG, has several advantages over free AMPs: 1) RPG improves the potency of AMPs by more than 9700-fold, 2) RPG presents a safe therapeutic profile, demonstrating stability in serum, efficacy under physiological conditions, and resistance to protease cleavage, and 3) the ePVX scaffold enables the multivalent tagging of different AMPs, achieving enhanced broader-spectrum targeting at lower concentrations.

The core foundation of this work enables multivalent tagging and scaffolding across high-aspect-ratio biocompatible nanostructures, exemplified here by ePVX. This provides a platform for different bioactive molecules to act synergistically and synchronously. In the context of the growing global burden of antimicrobial resistance and the urgent need for novel antibacterial strategies, this platform establishes a framework for deploying synergistic, resistance-proof therapeutics,^72^ providing a potent RPG in the fight against multidrug-resistant pathogens.

## Methods

### Plasmid Construction

Plasmid p104 carries a full-length PVX genome that closely resembles GenBank accession number M95516.1, with few mutations, and is flanked by the constitutively active cauliflower mosaic virus promoter (CaMV) 35S and terminator. The complete sequence of the plasmid DNA p104 containing the virus has been verified through Oxford Nanopore sequencing (SQK-LSK109, Oxford Nanopore, Oxford, UK) using the GridION sequencer (FLO-MIN106D). To introduce SpyCatcher003 at the N-terminal open reading frame (ORF) of the virus coat protein, a synthetic gBlock fragment was directly ordered containing a plant codon-optimized sequence for SpyCatcher003 (1VTT.37RE.AHT113), a short linker sequence (GG_2_S), and a ribosomal skipping peptide (NFDLLKLAGDVESNPG) from Foot-and-Mouth Disease Virus (FMDV). This fragment was flanked by 5′ *Xma*I and 3′ *Spe*I restriction sites for subsequent cloning into the *Xma*I/*Spe*I-digested p104 vector. For the expression of AMP variants, IDR-1018 (VRLIVAVRIWRR), 1002 (VQRWLIVWRIRK), IDR-3002 (ILVRWIRWRIQW)—see Supplementary Note 1 for a brief description of the peptides—they were directly cloned into the pSUMO2 vector (Addgene #52259) at the 5′ *Bam*HI and 3′ *Not*I sites, as a part of the commercial service provided by GenScript Biotech (Singapore). All AMPs were expressed with an N-terminus hexahistidine tag (G-HHHHHH), the Small Ubiquitin-like Modifier (SUMO) domain, followed by the respective AMP sequence, a short stretch of linker (GGGS)_3_, and the SpyTag003 (RGVPHIVMVDAYKRYK) at the C-terminus. All the constructs were verified by Sanger sequencing (King Abdullah University of Science and Technology Bioscience Core Lab), and the oligonucleotides were purchased from Integrated DNA Technologies (IDT, Leuven, Belgium).

### Nicotiana Benthamiana Inoculation

Tobacco plants (*Nicotiana benthamiana*) were grown in a greenhouse at 28 °C with 70% humidity under a 16-h light and 8-h dark cycle. The strain GV3101 (pSoup) of *A. tumefaciens* was electroporated (Bio-Rad Gene Pulser XCell Electroporation System, Hercules, CA) with p104 plasmid DNA encoding SpyCatcher003 and incubated at 28 °C for 48 h in Luria–Bertani medium supplemented with kanamycin (50 mg L^–1^), rifampin (25 mg L^–1^) and gentamycin (25 mg L^–1^). Cells were pelleted by centrifugation at 5000 × *g* for 10 min, then resuspended in MMA buffer containing 10 mM MES (2-[N-morpholino]-ethanesulfonic acid (Sigma-Aldrich, Cat. no. D134406), 10 mM MgCl_2_, and 100 μM acetosyringone (Sigma-Aldrich, Cat. no. 475893) to achieve a final OD_600_ of 0.5, followed by incubation of the bacterial suspension at 28 °C with gentle shaking for 3–4 h. Four-week-old plants were infiltrated with the bacterial strains on the abaxial side of *Nicotiana benthamiana* leaves using a 1 mL syringe to ensure complete leaf coverage. The infiltrated plants were then grown under greenhouse conditions until further analysis. Symptomatic tissues (which typically develop after 2 weeks) and the equivalent wild-type PVX-inoculated controls were harvested, flash-frozen in liquid nitrogen, and stored at −80 °C until further use.

### Protein Purification and Conjugation

The wild-type PVX accumulated to high titers in *N. benthamiana* leaves and was readily purified by virion precipitation with polyethylene glycol (PEG 8000), as described previously by Chapman.^73^ Leaf powder was homogenized by the addition of 1–3­[w/v] ice-cold extraction buffer (50 mM KP buffer, pH 8.0, 0.2% [v/v] β-mercaptoethanol, 1% [v/v] Triton X-100). The homogenate was filtered through two layers of microcloth, followed by the dropwise addition of 8% (v/v) *n*-butanol with gentle swirling and incubation for 15 min at room temperature. This resulted in the accumulation of pigmented chlorophyll and coagulated debris in the upper organic phase. The mixture was centrifuged at 10,000 × *g* for 30 min at 12 °C, and the lightly pigmented aqueous phase was recovered and filtered again through two layers of microcloth. Viral particles were precipitated from the filtered aqueous phase by adding PEG 8000 (Fisher Scientific, UK, Cat. no. 155383) to a final concentration of 4% (v/v) and incubation on ice for 15 min with occasional mixing. The precipitated viral particles were sedimented as a beige pellet by centrifugation at 10,000 × *g* for 15 min at 4 °C. The viral pellet was dissolved in 10 mM of KP buffer and centrifuged at 10,000 × *g* for 15 min at 4 °C to remove the residual pigmented material. The resulting supernatant, containing viral particles, was further saturated with 5 M NaCl to a final concentration of 17.5% (v/v) and 20% (w/v) PEG 8000 to a final concentration of 4% (v/v), followed by incubation on ice for 15 min. The white viral pellet, free of residual pigments, was recovered by centrifugation at 10,000 × *g* for 15 min at 4 °C. After complete removal of the supernatant, the pellet was resuspended in 10 mM KP buffer and stored at 4 °C until further use.

The SpyCatchered-PVX viral particles were purified from frozen symptomatic leaves using a protocol modified from Lee et al., and coworkers.^36^ All the harvested leaves were homogenized immediately in 1/3­[w/v] chilled extraction buffer (50 mM potassium phosphate, 10 mM EDTA, 10% [v/v] ethanol, 1% [v/v] β-mercaptoethanol, pH 7.2) at 4 °C until a slurry was formed. The slurry was passed through a microcloth and centrifuged at 7,800 × *g* for 20 min at 4 °C. The clear homogenate constituting the decanted supernatant was treated with a strong detergent, 1% (v/v) Triton-X 100, and gently stirred at 4 °C for 1 h. The preparation was centrifuged at 5,500 × *g* for 20 min at 4 °C, and the resulting clarified supernatant was treated with 1/5 of both [w/v] 1 M NaCl and 20% [w/v] PEG8000 (Fisher Scientific, UK, Cat. no. 155383), stirred for 1 h at 4 °C, and then incubated for another 1 h at room temperature. The viral particles were sedimented as a beige or white pellet at the bottom of the tube by centrifugation at 7,800 × *g* for 10 min at 4 °C. The viral pellet was resuspended in 0.05 M phosphate buffer (pH 8.0) with 1% [v/v] Triton X-100, the tubes were further rinsed with the same buffer, and the mixture was clarified by centrifugation at 7,800 × *g* for 10 min at 4 °C. The purified viral particles in the supernatant were buffer exchanged into 0.05 M potassium phosphate, concentrated using centrifugal filters with a 100-kDa NMWL membrane (Millipore, USA), and stored at 4 °C until use. Alternatively, a sucrose gradient of 10–50% was prepared using the automated Gradient Master (Biocomp Instruments, Model 153), overlaid with the purified viral particles, and centrifuged for 3 h at 194,000 × *g* in an SW55 Ti rotor (Beckman Coulter). Approximately 500 μL of fractions were collected, and the presence of viral particles was assessed using immunoblotting. Only the positive fractions were pooled, dialyzed against 0.05 M phosphate buffer, concentrated using centrifugal filters with a 100-kDa NMWL membrane (Millipore, USA), and stored at 4 °C until use.

All the SpyTagged AMPs were expressed in BL21 (DE3)-Rosetta *Escherichia coli* (Merck KGaA, Darmstadt, Germany) and purified using our in-house optimized peptide purification protocol.^10,74^ Briefly, cells were grown to an OD_600_ of 0.6, induced with 2.5 mM isopropyl-β-D-thiogalactopyranoside (IPTG; Gold Biotechnology, USA; Cat. no. 12481C100) and grown further at 37 °C for 4 h. Cells were harvested and lysed in the presence of Complete EDTA-free protease inhibitor cocktail tablet/50 mL (Roche, UK). The lysates were clarified by centrifugation at 10,000 x *g* for 1 h at 4 °C, filtered through a 0.45-μm membrane (Thermo Fisher Scientific, USA); the SUMO-AMPs were purified using a 5 mL HisTrap HP column (GE Healthcare Biosciences) and concentrated using centrifugal filters with a membrane NMWL of 10 kDa (Millipore, USA). The fused SUMO domain was cleaved using the orthogonal yeast SUMO protease Ulp1 (purified in-house from expression plasmid pFGET19_Ulp1 procured from Addgene #64697), and the released SpyTagged AMPs were freeze-dried (Labconco CentriVap −105 °C trap temperature ), resuspended in size-exclusion chromatography (SEC) buffer (5% [v/v] HPLC-grade CH_3_CN, 0.01 M HCl, and 150 mM NaCl), and clarified by centrifugation at 10,000 × *g* maintained at room temperature for 10 min. The clean supernatant containing released, tagged peptides was loaded into a SEC-buffer-pre-equilibrated Superdex 30 Increase 3.2/300 (GE Healthcare Biosciences) by manual injection via a 1.5 mL loop and eluted at a flow rate of 0.01 mL/min, with UV monitoring at 215/280 nm. Fractions containing the peptides devoid of the SUMO domain and protease were analyzed by immunoblotting, collected, subjected to freeze-drying, and stored at −20 °C until use.

Purified PVX-SpyCatcher003 rod-shaped nanoparticles were incubated with a 2-fold molar excess of either SpyTagged AMPx, SpyTagged AMPy, or both (1:1) at 22 °C for 3 h to generate homotypic and heterotypic particles (mosaic), respectively. The PVX-SpyCatcher003-Mosaic (RPG 2.0) particles were then passed through centrifugal filters with a membrane NMWL of 100 kDa (Millipore, USA) and stored at 4 °C until used for further experiments.

### Proteins/Peptides Detection and Quantification

For PVX analysis, viral particles were denatured by incubation at 98 °C for 5 min, and the released genomic RNA was used as a template to reverse transcribe using the SuperScript First-Strand Synthesis System (Invitrogen, Carlsbad, CA, USA) to generate cDNA. The resulting cDNA was subjected to PCR amplification using high-fidelity Phusion DNA polymerase (New England Biolabs, USA) and primers 5′- GCCATTGCCGATCTCAAGCCAC – 3′ and 5′- GCTACTATGGCACGGGCTGTAC – 3′, which flank the amino-terminal region of the PVX coat protein. The PCR products were separated on 1% [w/v] agarose gels, stained with Biotium GelRed (Fisher Scientific, Cat. No. NC9524151), and images were acquired using a ChemiDoc MP system (Image Lab Version 6.0.1, BioRad). The concentration of viral particles was determined using the Bradford assay, with a bovine serum albumin (BSA) standard curve, which served as a reference for protein quantification. For peptide quantification, the concentration was measured using the Pierce BCA Protein Assay Kit (Thermo Fisher Scientific, Cat. No. 23227).

For immunodetection of SpyCatchered viral particles at the protein level, the purified viruses were denatured by heating at 95 °C for 5 min, subsequently resolved on 12% NuPAGE gels (Thermo Fisher Scientific, CA, USA), and transferred to a 0.45 μm polyvinylidene difluoride membrane (PVDF, Amersham Hybond-P; GE Healthcare Life Sciences). SpyTagged peptides were resolved on 12% Tricine–SDS–PAGE gels alongside the Spectra Multicolor Low Range Protein Ladder (Thermo Fisher Scientific) and transferred to PVDF membrane. Primary antibodies used in this study included anti-SpyCatcher human/rabbit IgG antibody (Clone no. AbD41909 kg, BioRad, 1:4000), monoclonal anti-SpyTag human/rabbit IgG antibody (Clone no. AbD5176 kg, BioRad, 1:4000). The HRP-conjugated goat antirabbit IgG (Abcam, ab205718) was used as the secondary antibody at a 1:5000 dilution. Immunoblotting bands were detected using an enhanced chemiluminescence reagent (ECL kit; Amersham Pharmacia Biotech), and blot images were captured with an ImageQuant LAS 4000 (Version 1.0, GE Healthcare Biosciences).

SpyCatcher decoration was estimated via densitometric analysis of SDS-PAGE bands using ImageJ software. This semiquantitative approach provides relative abundance values suitable for normalization. Peptide loading was assumed to be 100% since it is practically difficult to resolve loaded from unloaded coat protein units (difference of approximately 10% in size, which is difficult to resolve on SDS-PAGE). It is important to note that 100% loading assumption gives the lowest fold change in efficacy.

### Scanning Electron Microscopy

The untreated and treated bacterial cells in Cation-adjusted Müller-Hinton broth (Ca-MHB; Millipore, Cat. no. 90922) were transferred on Poly-l-Lysine- (Sigma-Aldrich, Cat. no. P6282) coated coverslips, and the media was aspirated before overnight fixation at 4 °C using modified Karnovsky’s fixative (2.5% [w/v] glutaraldehyde and 2% [w/v] paraformaldehyde in 0.1 M sodium cacodylate buffer, pH 7.4) procured from Electron Microscopy Sciences, USA, Cat. no. 15949. The fixed specimens were postfixed for 1 h in the dark with 1% (w/v) osmium tetroxide and 1.5% (w/v) potassium ferrocyanide prepared in 0.1 M sodium cacodylate buffer, followed by three washes with distilled water (15 min each). Samples were then dehydrated through a graded ethanol series (30%, 50%, 70%, 90%, and 100%) and dried using a critical point dryer (CPD300, Leica, Germany). The dehydrated specimens were mounted on SEM stubs and sputter-coated with a 10 nm platinum layer (K575X Sputter Coater, Quorum). Imaging was performed using FEI Nova Nano 630 SEM (Thermo Fisher, Oregon, USA) operating at 3 kV and equipped with Everhart–Thornley (ETD) and through-the-lens (TLD) detectors.

### Minimal Inhibitory Concentration

The minimum inhibitory concentrations (MICs) of the antibacterial agents were determined using a liquid growth inhibition assay in 96-well microtiter plates, established in our laboratory and described previously.^10,74^ The pathogenic bacterial strains used in the study included methicillin-resistant *Staphylococcus aureus* USA300, carbapenem-resistant *Escherichia coli* *PI7* (a New Delhi metallo-β-lactamase-positive strain previously isolated from municipal wastewater in Saudi Arabia), *Acinetobacter Junii* DSMZ 14968, extended-spectrum β-lactamase-producing *Klebsiella pneumoniae* ATCC 700603, *Enterobacter faecalis* ATCC 29212, and *Pseudomonas aeruginosa* ATCC 9027. In the case of *E. coli* *PI7*, the strain was grown in Luria–Bertani media (LB; BD Difco Cat. no. 244620) supplemented with 8 μg mL^–1^ Meropenem (Sigma-Aldrich, Cat. no. PHR1772). Methicillin-resistant *S. aureus* was cultured in tryptic soy broth (TSB; Difco, Detroit) containing 10 μg mL^–1^ chloramphenicol (Sigma-Aldrich, Cat. no. C0378), while the remaining strains were cultured in LB media without any antibiotic supplement. For the MIC assay, pathogenic bacterial cells were cultured overnight in their respective media at 37 °C, and the next day, diluted to grow until the OD_600_ of 0.5. Subsequently, 90 μL of the bacterial suspension prepared in Ca-MHB (Millipore, Cat. no. 90922) was added to the 96-well microtiter plate (Corning), followed by the addition of 10 μL of the respective bacterial killing agent. Plates were incubated for 24 h at 37 °C, and bacterial growth was quantified by measuring OD_600_ using a Tecan Infinite Nanoquant 200 Microplate Reader (Tecan Austria, GmbH).

### Antibiotics and Synthetic Peptides

For *in vitro* studies, vancomycin hydrochloride (Sigma-Aldrich, Cat. no. PHR1732), colistin sulfate (Sigma-Aldrich, Cat. no. C4461), cefiderocol (Sigma-Aldrich, Cat. no. SBR00041), and tigecycline (Thermo Fisher Scientific, Cat. no. 463062500) were used. All antibiotics were reconstituted in their respective sterile solvents according to the manufacturers’ instructions and stored at −20 °C until further use. High-performance liquid chromatography (HPLC)-purified synthetic peptides (AMPx, AMPy, AMPz) were commercially procured from GenScript (Singapore) and dissolved in endotoxin-free, sterile water for *in vitro* experiments.

### Propidium Iodide Uptake Assay

The fluorescent probe propidium iodide (PI; Molecular Probes, Invitrogen, USA) was used to assess bacterial membrane permeabilization induced by the antibacterial agent. Methicillin-resistant *S. aureus* cells were cultured to the mid-logarithmic growth phase, diluted to 1 × 10^8^ CFU/mL in Ca-MHB, and exposed to antimicrobial agents. Bacterial samples were then treated with PI at a final concentration of 1 μg/mL and incubated for 30 min in the dark at room temperature. The relative quantification of PI uptake (percent influx) was performed with a BD LSRFortessa Cell Analyzer (BD FACSDiva Software, v6.2, BD Biosciences, San Jose, CA, USA), and the data were analyzed with FlowJo 10.6.2 software (BD Biosciences).

### NPN Outer Membrane Permeabilization Assay

To assess outer membrane permeabilization, we employed the 1-*N*-phenylnaphthylamine (NPN) uptake assay as previously described by Hancock and Wong.^11^ NPN is a hydrophobic, uncharged fluorescent probe with low intrinsic fluorescence in aqueous environments that partitions into the hydrophobic core of destabilized membranes, producing a marked increase in emission intensity. Bacterial cultures were grown to OD_600_ = 0.5, pelleted by centrifugation, and resuspended in 5 mM HEPES buffer (pH 7.2) supplemented with 5 mM glucose to a final density of 0.4 OD/mL. RPG 2.0 was prepared in endotoxin-free sterile water and added to a 96-well plate at MIC. NPN (10 μL, 0.5 mM; Sigma-Aldrich, Cat. no. 104043) was added in the dark to prevent photobleaching, followed by 90 μL of bacterial suspension. Fluorescence readings were taken immediately and recorded every minute for 30 min (λ_ex_ = 340 nm, λ_em_ = 405 nm) using a TECAN Infinite 200 PRO microplate reader (Tecan i-control 2, v2.0.10.0, Austria GmbH). Controls included HEPES alone, HEPES with NPN, HEPES with bacteria, and HEPES with bacteria and NPN. All experiments were performed in triplicate.

### RPG 2.0 Toxicity Assessment in Primary Dermal Fibroblasts

Primary human dermal fibroblasts (HDFs) isolated from neonatal foreskin were purchased from Gibco (Cat. no. C0045C) and cultured in DMEM with high glucose (Gibco, Cat. no. 11965092), supplemented with 10% heat-inactivated fetal bovine serum (Gibco, Cat. no. 10437028) and 1% penicillin–streptomycin (Gibco, Cat. no. 15140122). Cells were maintained at 37 °C in a humidified atmosphere containing 5% CO_2_ and used between passages 3–8.

Fibroblasts were seeded at a density of 1 × 10^4^ cells per well in 96-well plates. The purified RPG 2.0 was then added at its respective minimum inhibitory concentration. After 2 days of incubation, cells were stained with 2 μM calcein AM and 4 μM ethidium homodimer-1 (LIVE/DEAD Viability/Cytotoxicity Kit for Mammalian Cells, Thermo Fisher Scientific, Cat. no. L3224) for 40 min in the dark. Prior to imaging, the staining solution was removed and replaced with fresh PBS. Stained cells were visualized using an inverted confocal microscope (Carl Zeiss LSM710, Germany).

Immunostaining was performed after 84 h of incubation of purified RPG 2.0 with cells, as described previously^10,74^ (Chaudhary et al). Briefly, cells were fixed with 4% (w/v) paraformaldehyde for 30 min and permeabilized with cold cytoskeleton buffer (3 mM MgCl_2_, 300 mM sucrose, and 0.5% [v/v] Triton X-100 in PBS) for 5 min. The cells were then blocked with buffer containing 5% (v/v) FBS, 0.1% (v/v) Tween-20, and 0.02% (w/v) sodium azide in PBS for 30 min at 37 °C. Then, F-actin was stained with rhodamine–phalloidin (Thermo Fisher Scientific, Cat. no. R415; 1:300) for 1 h at room temperature in the dark, followed by three washes with PBS. Nuclei were counterstained with NucBlue Fixed Cell ReadyProbes Reagent (DAPI; Thermo Fisher Scientific, Cat. no. R37606; 1:2000) for 5 min, and excess stain was removed by washing with PBS. Stained cells were visualized using a laser scanning confocal microscope (Leica Stellaris, Leica Application Suite X, Germany).

### Hemolysis Assay

The hemolytic activity of RPG against human erythrocytes was evaluated following the protocol described by Oddo and Hansen^75^ with minor modifications. One milliliter of freshly collected whole blood was transferred to a 15 mL centrifuge tube and centrifuged at 800 g for 8 min to collect the bottom RBC layer. The RBCs were washed 3 times at 1× phosphate-buffered saline (PBS, pH 7.4). The washed erythrocytes were then diluted in PBS to yield a 0.5% (v/v) working suspension (40 μL of packed erythrocytes per 8 mL of PBS).

For the hemolysis assay, serial dilutions of RPGx, RPGy, and RPG 2.0 were prepared in 1× PBS. In a sterile 96-well plate, 20 μL of each nanoparticle dilution was combined with 180 μL of the 0.5% erythrocyte suspension, yielding a final assay volume of 200 μL per well. Wells containing erythrocyte suspension mixed with 0.1% Triton X-100 served as the positive control to define 100% hemolysis, while wells containing erythrocytes in PBS alone served as the negative control to define baseline (0%) hemolysis. Free AMPx and AMPy were also included at equivalent concentrations for comparison. Following incubation at 37 °C for 1 h, intact erythrocytes and cellular debris were pelleted by centrifugation, and 60 μL of the clarified supernatant from each well was transferred to a fresh 96-well plate. The absorbance of released hemoglobin was measured at 414 nm using a microplate reader. The percentage hemolysis for each sample was calculated according to the following formula:

$$\mathrm{Hemolysis}\;\left(\%\right)=\frac{{\mathrm{OD}}_{570}\left(\mathrm{sample}\right)-{\mathrm{OD}}_{570}\left(\mathrm{negative control}\right)}{{\mathrm{OD}}_{570}\left(\mathrm{positive control}\right)-{\mathrm{OD}}_{570}\left(\mathrm{negative control}\right)}\times 100\%$$

### Charge Neutralization Study (Heparin)

To validate that enhanced antimicrobial activity is charge-dependent, RPG 2.0 (6.25 μg/mL) was preincubated with increasing concentrations of heparin sodium (0–51.2 ng/mL; Sigma-Aldrich, Cat. no. H3149) in MHB for 1 h at 37 °C, allowing electrostatic binding and charge neutralization. Following incubation, untreated *E. coli* *PI7* was added and MIC was determined as previously described. Heparin control (toxicity control) and untreated RPGx controls were included in parallel.

### Zeta Potential Measurements

Zeta potential measurements were performed using a Litesizer 501 Particle Analyzer (Anton Paar, Graz, Austria). Purified viral particles were diluted in 0.1 M potassium phosphate (KP) buffer, pH 8.0, to final concentrations of 31.5 nM (wtPVX), 24 nM (ePVX), and 22.5 nM (RPGx). A sample volume of 50 μL was loaded into disposable Univette low-volume cuvettes. Measurements were conducted at 25 °C with automatic voltage optimization. Data acquisition and analysis were performed using Kalliope software (Anton Paar).

### Atomic Force Microscopy

Viral particles in 100 mM potassium phosphate buffer (pH 8) at concentrations of 1.58 nM (wtPVX), 1.2 nM (ePVX), and 1.13 nM (RPGx) were used for imaging. A 10 μL aliquot of the sample was deposited onto a freshly cleaved mica surface (V1 grade, Ted Pella, Inc.). After 10 s of incubation, the mica surface was rinsed three times with 200 μL of deionized water and air-dried for 1 h in a vacuum desiccator. AFM measurements were performed using a Bruker Dimension Icon AFM in tapping mode with an FESPA-V2 probe (Bruker) and cantilevers with a frequency of 50–100 kHz and a spring constant of 2.8 N m^–1^. Images were collected with a resolution of 256 × 256 pixels. Height measurements were extracted from topography images using Gwyddion software, with values reported as mean ± standard deviation.

### Protein Identification by LC-MS/MS

Mass spectrometry analysis to confirm the formation of SpyCatcher-SpyTag conjugate was conducted with an Orbitrap Exploris 480 mass spectrometer coupled with Vanquish Neo UHPLC system (Thermo Scientific). A mixture of PVX-CP-SpyCatcher and AMP-SpyTag after conjugation reaction was digested with trypsin following in-gel digestion protocol.^76^ Approximately 200 ng of the digested peptide mixture was injected with autosampler, and separation was performed at 40 °C on a 75 μm × 500 mm C18 column (EASY-Spray PepMap NEO 1500 bar, Thermo Scientific) with a gradient of acetonitrile ranging from 2% to 60% in the presence of 0.1% formic acid over the course of 60 min. Eluted peptides were subsequently ionized and subjected to separation by high-field asymmetric waveform ion mobility spectrometry (FAIMS) with the compensation voltage (CV) of −45 V, and introduced to the mass spectrometer for the analysis utilizing higher-energy collisional dissociation (HCD) with the normalized collision energy of 26%. Survey scans were performed at 120,000 resolution, while fragment detection was at 15,000 resolution, both in the Orbitrap. Data acquisition was conducted as data-dependent acquisition (DDA) while the mass spectrometer was operated in positive mode. Data analysis was carried out with Mascot Server 3.0 software (Matrix Science).

### Negative Staining of Virus Particles for Transmission Electron Microscopy

For negative staining, 5 μL of virus suspension was deposited onto copper grids coated with a continuous carbon film (Electron Microscopy Sciences) and allowed to adsorb for 5 min at room temperature. Excess liquid was gently removed by blotting with filter paper, and the grids were washed three times with Milli-Q water.

The grids were then negatively stained with a 1% (w/v) aqueous uranyl acetate solution for 30 s. After blotting off the stain, the staining step was repeated once using fresh uranyl acetate for an additional 30 s. Following the final blotting step, the grids were air-dried completely and examined using a transmission electron microscope (Talos F200X, Thermofischer) operated at an accelerating voltage of 200 kV.

## Supplementary Material



## Notes

Supplementary Note 1 on AMP production; Supplementary Note 2 on zeta potential; Supplementary Note 3 on AFM analysis; Supplementary figures and tables; DNA constructs used in this study (PDF)
