# Supplementary material for: Multivalent Display of Antimicrobial Peptides on Plant Virus Scaffolds Enhances Killing of Drug-Resistant Bacteria
Source: ACS Nano. 2026 Jun 16;20(35):24318–33. doi: 10.1021/acsnano.6c03117 (PMC13644466; doi:10.1021/acsnano.6c03117)
Supplement: Supplementary file 1 [file nn6c03117_si_001.pdf]

Multivalent Display of Antimicrobial Peptides on Plant Virus Scaffolds Enhances Killing of Drug-Resistant Bacteria

Ahmed Ghouneimy[a], Shahid Chaudhary[a], Muhammad Tehseen[b], Ahmed Saleh[a], Maged Serag[c], Maya Ayach[d], Ebtihaj Bukhari[d], Maazallah Masood[a], Kosuke Sakashita[e], Pei-Ying Hong[f], Satoshi Habuchi[c], Samir M. Hamdan[b], and Magdy Mahfouz[a] ✉

[a] *Laboratory for Genome Engineering and Synthetic Biology, Division of Biomedical Sciences, 4700 King Abdullah University of Science and Technology, Thuwal 23955-6900, Saudi Arabia.*

[b] *Laboratory of DNA Replication and Recombination, Division of Biomedical Sciences and Engineering, King Abdullah University of Science and Technology (KAUST), Thuwal, 23955-6900, Saudi Arabia*

[c] *Molecular Imaging and Microscopy Lab, Division of Biomedical Sciences and Engineering, King Abdullah University of Science and Technology (KAUST), Thuwal, 23955-6900, Saudi Arabia*

[d] *Imaging & Characterization Core Laboratory, King Abdullah University of Science and Technology (KAUST), Thuwal, 23955-6900, Kingdom of Saudi Arabia*

[e] *Analytical Chemistry Core Lab, King Abdullah University of Science and Technology (KAUST), Thuwal, 23955-6900, Kingdom of Saudi Arabia*

[f] *Water Desalination and Reuse Center, Division of Biological and Environmental Sciences and Engineering, King Abdullah University of Science and Technology (KAUST), Thuwal, 23955-6900, Saudi Arabia*

✉ Correspondence: Magdy M. Mahfouz (magdy.mahfouz@kaust.edu.sa)

Keywords**:** Multidrug resistance, antimicrobial peptides, bioproduction, PVX, Plant Virus, MRSA.

Supplementary information

Supplementary Notes

Supplementary Note 1

For AMP production, we designed expression constructs optimized for *E. coli*. Because free AMPs[1] are cytotoxic to bacterial hosts, peptides were expressed as fusion proteins with an N-terminal SpyTag and a C-terminal His–SUMO tag, enhancing solubility and enabling purification. Western blotting using anti-SpyTag antibodies confirmed expression of the AMP fusion protein (Fig. S1). The AMP was then conjugated to the SpyCatcher-decorated PVX via covalent SpyTag–SpyCatcher ligation. The conjugation was confirmed by a characteristic molecular weight shift in SDS-PAGE and western blot using anti-SpyCatcher antibodies. Subsequent purification steps ensured the removal of unbound peptides (Fig. S1), yielding a virus–AMP conjugate ready for functional evaluation.

In this study we have used three peptides: IDR-1018 (AMPx), IDR-1002 (AMPy), Peptide 3002 (AMPz). IDR-1018 (VRLIVAVRIWRR) adopts a predominantly α-helical structure in membrane-mimetic environments but is conformationally flexible and can adopt a β-sheet state depending on microenvironment conditions[2,3]. It's a membranolytic peptide with antibiofilm and immunomodulatory properties[2-4]. IDR-1002 (VQRWLIVWRIRK) has been structurally investigated by NMR for three-dimensional structure determination[5] and was shown to adopt a predominantly α-helical conformation in membrane-mimetic environments, while remaining conformationally flexible or disordered in aqueous solution, consistent with host defense peptides[6]. 1002 is a peptide with good immunomodulatory activity in vitro and in vivo. Peptide 3002 (ILVRWIRWRIQW) is a relatively new peptide derived from IDR-1018 for antibiofilm activity towards S.aureus and exhibits immunomodulatory activity and works in vivo. It has not been structurally resolved at high resolution[7].

The small size of AMPs relative to the size of the ePVX coat protein makes it difficult to confirm conjugation using standard laboratory methods. Accordingly, we performed MS peptide sequencing to validate the conjugation. One potential modification to the current design is to reengineer ePVX by fusing SpyTag (4 kDa) to the coat protein and fusing SpyCatcher (15 kDa) to

the peptide. This design could simplify detection and quantification on the gel. Moreover, hybrid production of ePVX *in planta* and AMP in *E. coli* could limit mass production. An alternative design is to produce both parts in separate plant lines and fuse the scaffold to AMP by simple mixing of plant lysates.

Supplementary Note 2

Zeta potential measures the electrical potential at the slipping plane of the electrical double layer surrounding charged particles in solution, rather than the actual surface charge of the particle itself. When a charged particle is suspended in an aqueous medium, it attracts a tightly bound layer of counterions (Stern layer), followed by a diffuse layer of ions that extends into the bulk solution. The zeta potential reflects the net charge of this diffuse ionic atmosphere at the plane of shear (plane between ion layer and water), where the particle and its associated ion cloud move as a single unit during electrophoretic migration inside the Zeta potential measurement cell.

We initially observed a stepwise reduction in negative zeta potential from wtPVX (−9.31 mV) to ePVX (−3.09 mV) to RPGx (−1.21 mV), indicating a progressive shift toward charge neutralization, consistent with the sequential addition of positively charged moieties. However, the observation that RPGx remains slightly negative (−1.21 mV) despite conjugation with cationic AMPs can be explained by considering multiple factors that influence the measured zeta potential beyond the intrinsic surface charge of the nanoparticle. First, the original measurements were conducted in 0.1 M KP buffer, which provides a high concentration of counterions that electrostatically shield surface charges. Cationic groups on the AMPs attract anionic species from the buffer, forming a condensed counterion layer that reduces the net charge measured at the slipping plane. Second, at the measurement pH, ionizable groups on both the viral coat proteins and AMPs may not be fully protonated or deprotonated, further affecting the net charge distribution.

To address this directly, we repeated the zeta potential measurements under low ionic strength conditions (0.0005 M KP buffer) to minimize counterion shielding and reveal the intrinsic surface charge. Under these conditions, a clear positive charge trend emerged: wtPVX (mean = +0.47 mV), ePVX (mean = +28.6 mV), and RPGx (mean = +40.8 mV). These results confirm that the progressive functionalization from wtPVX to ePVX to RPGx does indeed confer increasingly

positive surface charge, which was masked in the original measurements by the high ionic strength of 0.1 M KP buffer.

Supplementary Note 3

Interpretation of AFM data requires careful consideration of inherent artifacts and the conditions under which measurements are obtained.

1. Height vs Width Measurements in AFM

In AFM imaging, height measurements are more accurate than lateral (length/width) dimensions. In contrast, lateral dimensions are subject to tip artifacts, where the finite size and shape of the AFM probe tip cause apparent broadening of narrow features. Consequently, we report height measurements as the primary dimensional descriptor for ePVX and RPGx.

2. Sample Flattening in Dry AFM

All AFM imaging in this study was performed on air-dried samples. Biological samples, particularly protein assemblies, are known to undergo significant structural perturbation when dried onto substrates (unfolding) and during scanning (deformation).

The flattening phenomenon arises from multiple factors: (1) loss of the hydration shell during drying, which normally provides structural support; (2) electrostatic and van der Waals interactions between the particle and the mica substrate, which can partially denature proteins at the contact interface. Importantly, the magnitude of flattening depends on the intrinsic mechanical properties of the particle (more rigid structures resist deformation better than flexible or loosely assembled structures).

3. Progressive Reduction in SpyCatcher-free Region Height: wtPVX → ePVX → RPGx

The observed trend in SpyCatcher-free region heights (ePVX (5.83 nm) > RPGx (3.84 nm)) reflects a gradient of mechanical compliance under the combined stresses of adhesion, drying, and repeated AFM tip scanning. This trend does not necessarily indicate spontaneous disassembly in solution, but rather differential susceptibility to mechanical perturbation under non-physiological conditions.

Individual particles vary in their SpyCatcher density (as shown in Fig. 7). Particles may have reduced inter-subunit cohesion due to disruption of native coat-coat interactions. The 5.83 nm height represents these vulnerable regions after experiencing drying and imaging.

For RPGx: The conjugation of cationic AMPs introduces a high density of positive charges on the viral surface. These high-density cationic peptides likely alter electrostatic interactions between coat protein subunits (native PVX capsid assembly is stabilized by networks of salt bridges and hydrogen bonds), and the addition of positively charged AMPs may disrupt these stabilizing

interactions, particularly if AMPs interact with negatively charged residues on adjacent coat proteins. The result is a more compliant structure that deforms more readily under stress. SpyCatcher-AMP-free regions of RPGx (3.84 nm) experience the greatest flattening, while AMP-decorated regions (14.95 nm) maintain greater height, possibly because the AMP clusters provide some mechanical resistance.

This mechanical fragility under AFM imaging conditions does not necessarily translate to instability under physiological conditions. The antimicrobial assays are performed in aqueous buffer at 37°C, where particles remain hydrated and are not subjected to substrate adhesion forces or mechanical probing.

4. High Standard Deviation in RPGx Decorated Region Height (±4.53 nm)

The height of RPGx decorated regions (14.95 ± 4.53 nm) exhibits a standard deviation nearly 10-fold greater than that of ePVX decorated regions (10.10 ± 0.53 nm). This variability likely arises from heterogeneous AMP distribution and clustering on the viral surface.

Supplementary figures

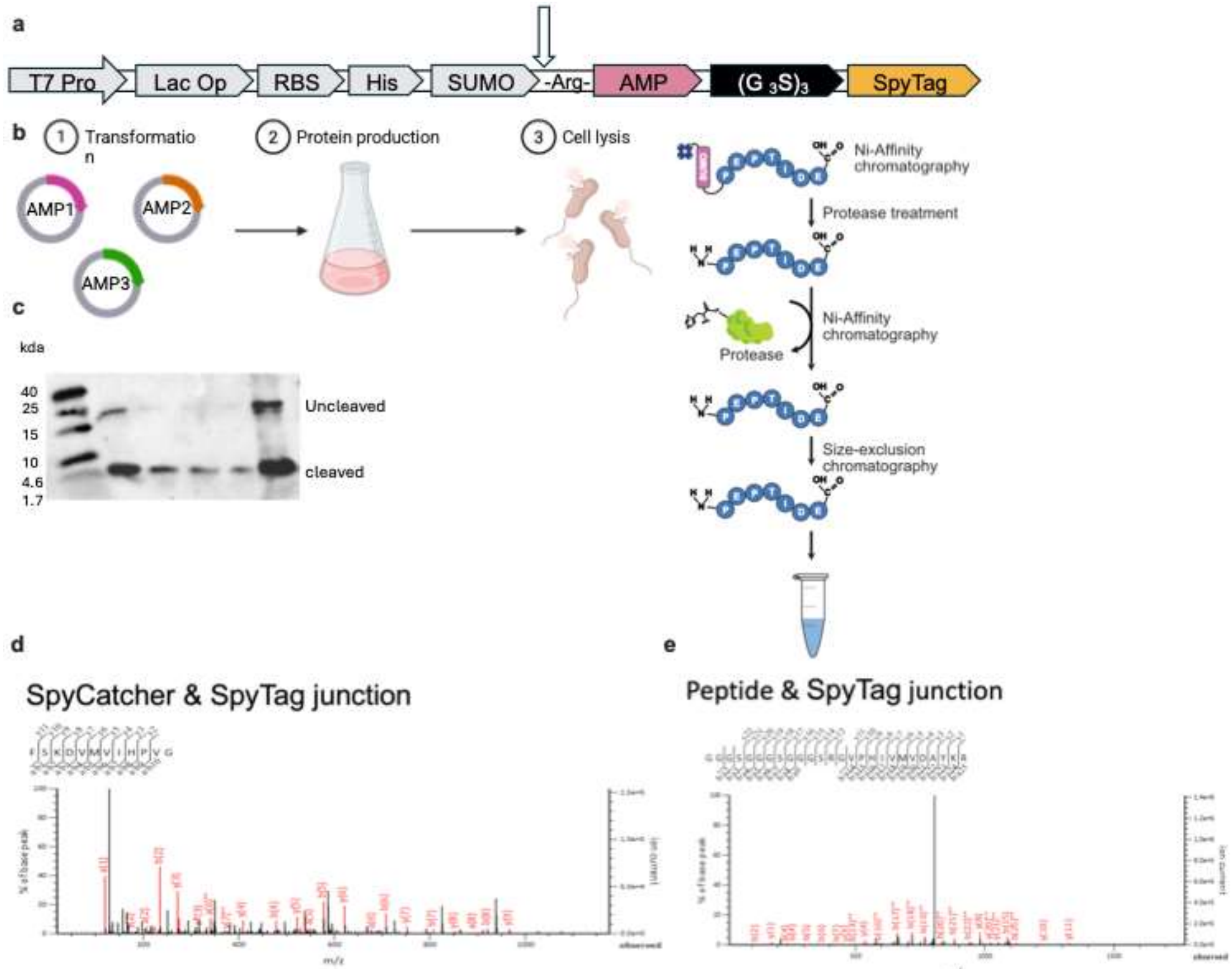

Figure S1 | Bacterial expression and purification of SpyTagged AMP and confirmation of tagging. a, AMP was expressed under a T7 promoter and the expression product contained His tag for purification and SUMO cleavage site to obtain native AMP. Additional Arginine was placed after SUMO cleavage site as it was shown to enhance cleavage efficiency. **b,** Plasmids were transformed into *E. coli*, cells were grown and then lysed chemically and mechanically. This was followed by Ni-affinity chromatography where His tag binds specifically to Ni-functionalized beads. The product was subjected to SUMO cleavage and then dried and resuspended in Acetonitrile buffer. This was followed by size exclusion chromatography. The final peptide was dried again and resuspended in water. **c,** Western blot with anti-SpyTag antibody showing cleaved and uncleaved AMPs. **d,** MS peptide sequencing of conjugated virus and peptides showing successful conjugation and e, part of the SpyTag and peptide junction. AMP, antimicrobial peptide; MS, mass spectrometry.

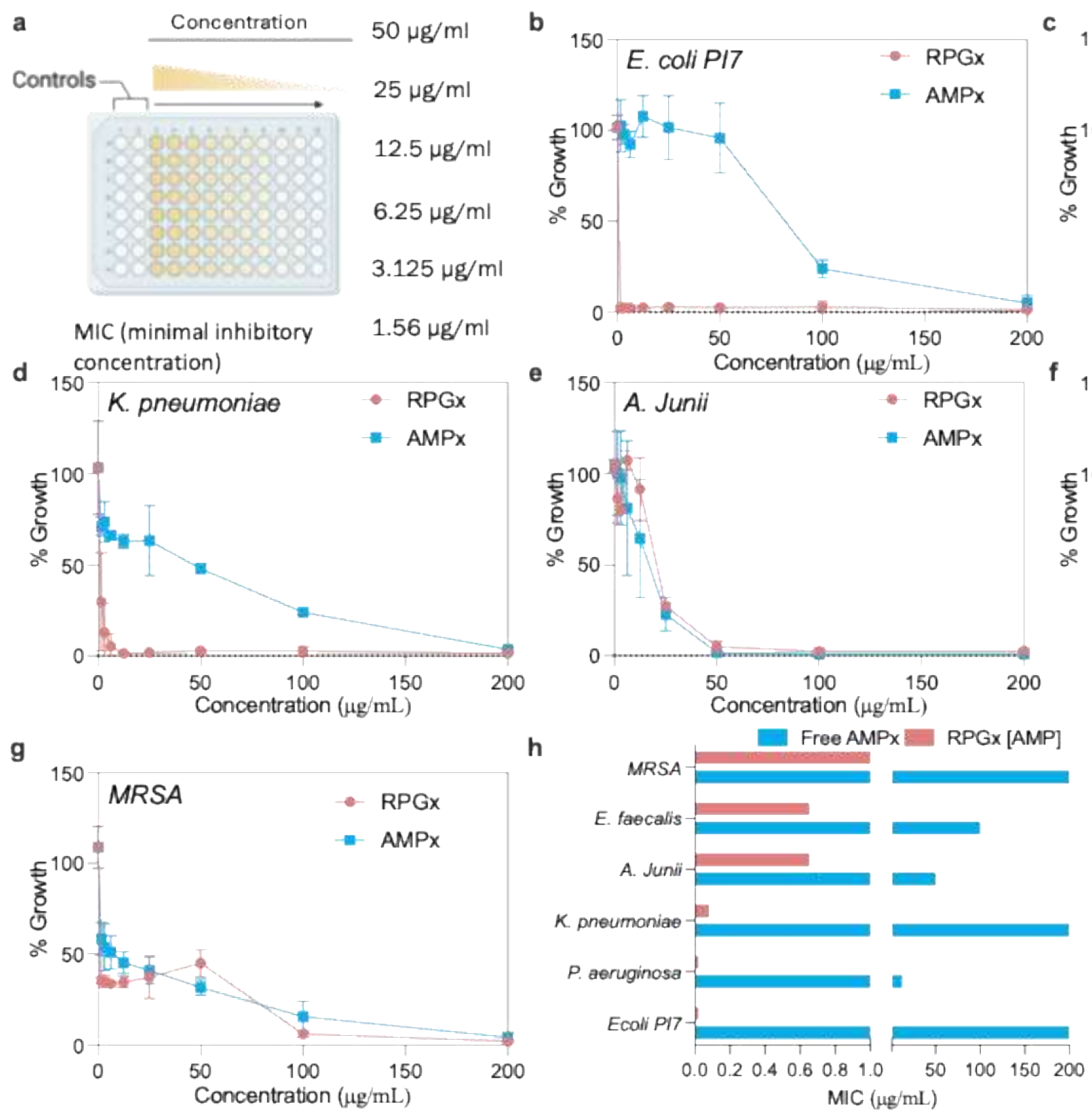

Figure S2 | MIC of RPGx and AMPx on MDR pathogens. a, schematic representation of the MIC experiment. **b-g,** MIC of RPGx and AMPx on **(b)** *E. coli PI7*, **(c)** *P. aeruginosa*, **(d)** *K. pneumoniae*, **(e)** *A. Junii*, **(f)** *E. faecalis*, and **(g)** *MRSA*. **h,** comparison of the amount of AMP required at MIC in free AMPx and conjugated AMPs (RPGx [AMP]). Here we quantified the MIC of AMPs on the virus in a head-to-head comparison with the MIC of free AMPx. All experiments are repeated three times independently with n = 3. RPG, Rod-based Peptide Grids; MIC, minimum inhibitory concentration; MDR, multidrug-resistant.

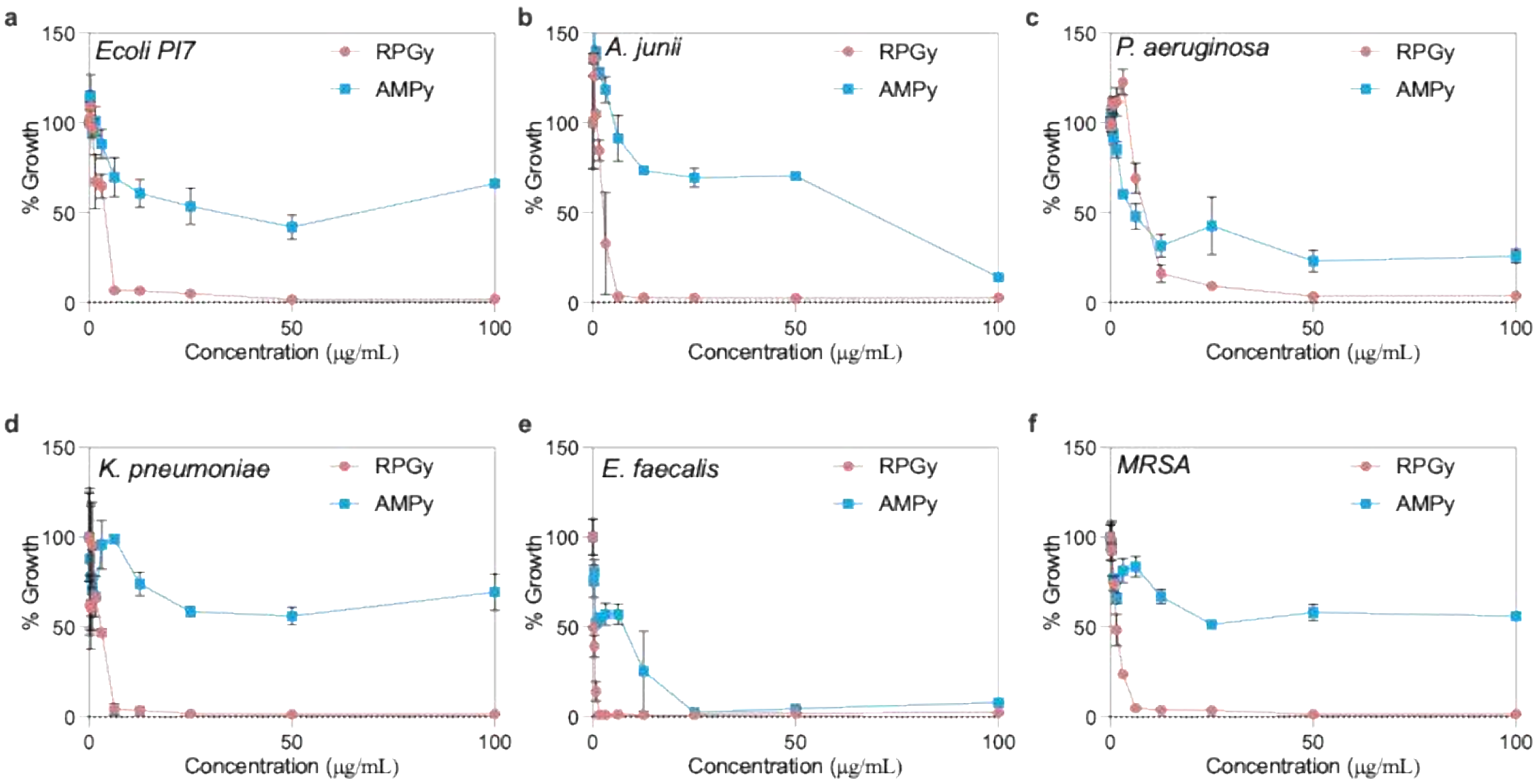

Figure S3 | MIC of RPGy. a-f, MIC of RPGy and AMPy on **(a)** *E.coli PI7*, **(b)** *P. aeruginosa*, **(c)** *K. pneumoniae*, **(d)** *A. Junii*, **(e)** *E. faecalis*, and **(f)** *MRSA*. All experiments are repeated three times independently with n = 3. RPG, Rod-based Peptide Grids; MIC, minimum inhibitory concentration; MRSA, methicillin-resistant Staphylococcus aureus.

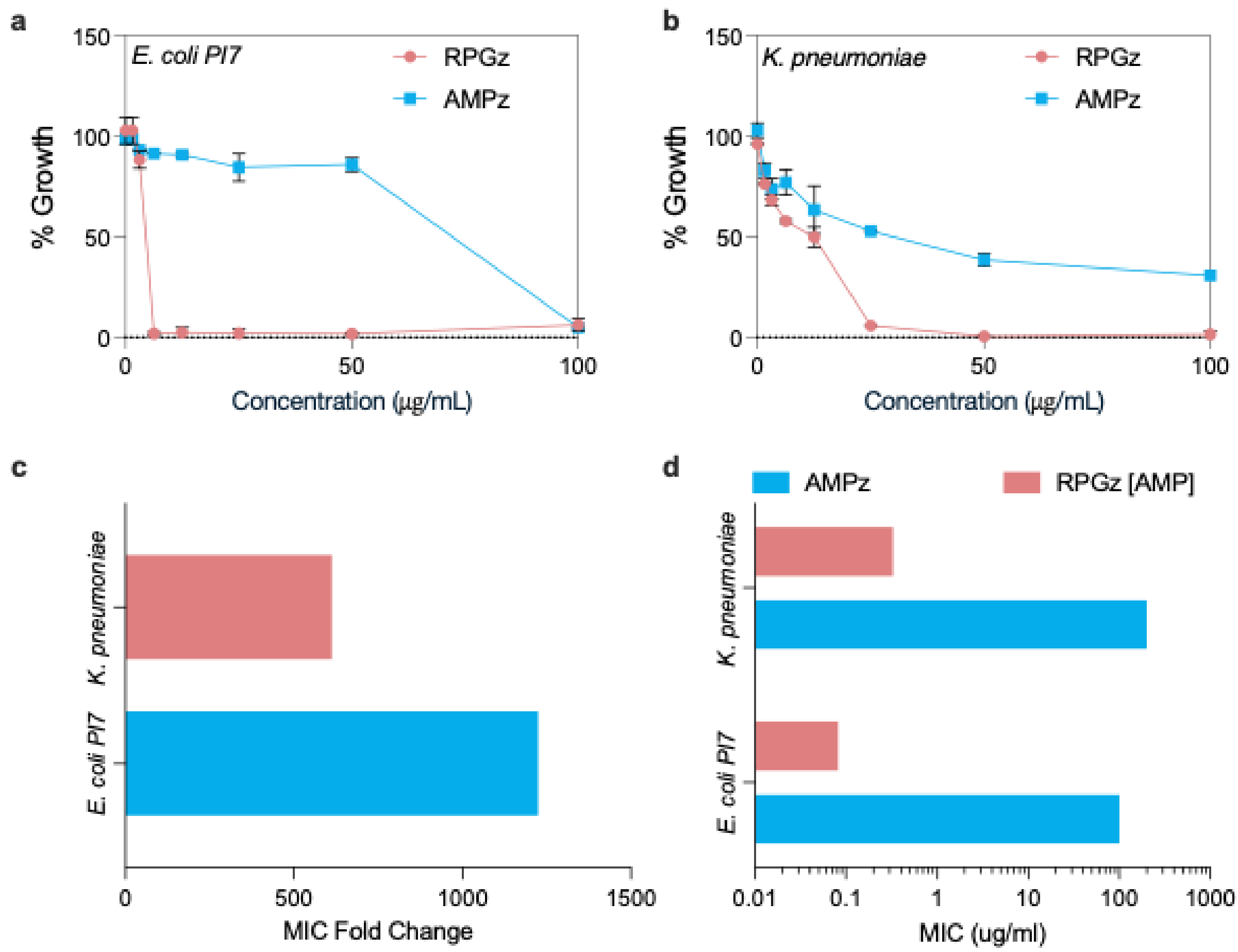

Figure S4 | MIC of RPGz. a-f, MIC of RPGz and AMPz on **(a)** *E.coli PI7* and **(b)** *K. pneumoniae*. **c,** fold change of MIC of conjugated AMPz relative to free AMPs. Fold change was calculated by dividing the MIC of free AMPz by the MIC of the actual concentration conjugated AMPz (AMPz present on the viral surface). **d,** comparison of the amount of AMP required at MIC in free AMPz and conjugated AMPs (RPGz [AMP]). Here we quantified the MIC of AMPs on the virus in a head-to-head comparison with the MIC of free AMPz. All experiments are repeated three times independently with n = 3. RPG, Rod-based Peptide Grids; MIC, minimum inhibitory concentration; MRSA, methicillin-resistant Staphylococcus aureus.

Table S1 | Minimum inhibitory concentrations (MICs) of RPGx and last-resort antibiotics against multidrug-resistant pathogens.

Pathogen	RPGx MIC (μg/mL)	Reference antibiotic	Antibiotic MIC (μg/mL)
Escherichia coli PI-7	3.125	Colistin	0.25
MRSA USA300 (*Staphylococcus aureus*)	1.5	Vancomycin	1.25
Klebsiella pneumoniae ATCC 700603	1.5	Tigecycline	1
Pseudomonas aeruginosa ATCC 9027	1.5	Cefiderocol	12.8

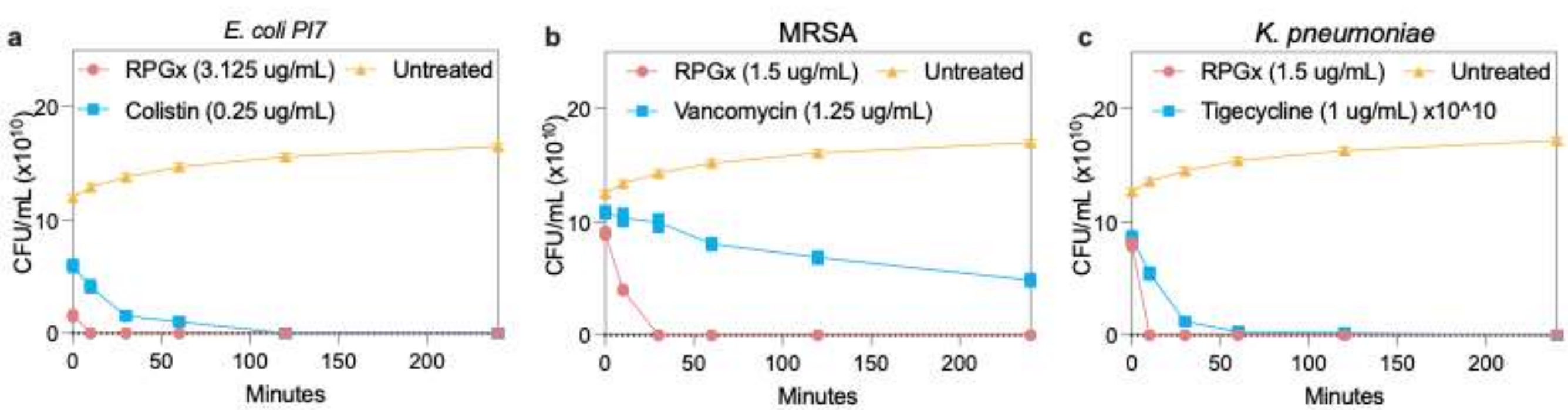

Figure S5 | Time-kill kinetics of RPGx against MDR pathogens. a–c, Time-kill curves comparing RPGx and last-resort antibiotics against an initial inoculum of 1×10^{11} CFU/mL of (a) *E. coli PI-7*, (b) MRSA, and (c) *K. pneumoniae*, in cation-adjusted Mueller-Hinton broth (Ca-MHB) at 37 °C. Each pathogen was exposed to RPGx at its MIC (3.125 μg/mL for E. coli PI-7; 1.5 μg/mL for MRSA and K. pneumoniae) or to its corresponding last-resort antibiotic at MIC:

Colistin (0.25 μg/mL) for E. coli PI-7, Vancomycin (1.25 μg/mL) for MRSA, and Tigecycline (1 μg/mL) for K. pneumoniae. Untreated bacteria served as growth controls All experiments are repeated three times independently with n = 3. MIC, minimum inhibitory concentration; RPG, Rod-based Peptide Grids.

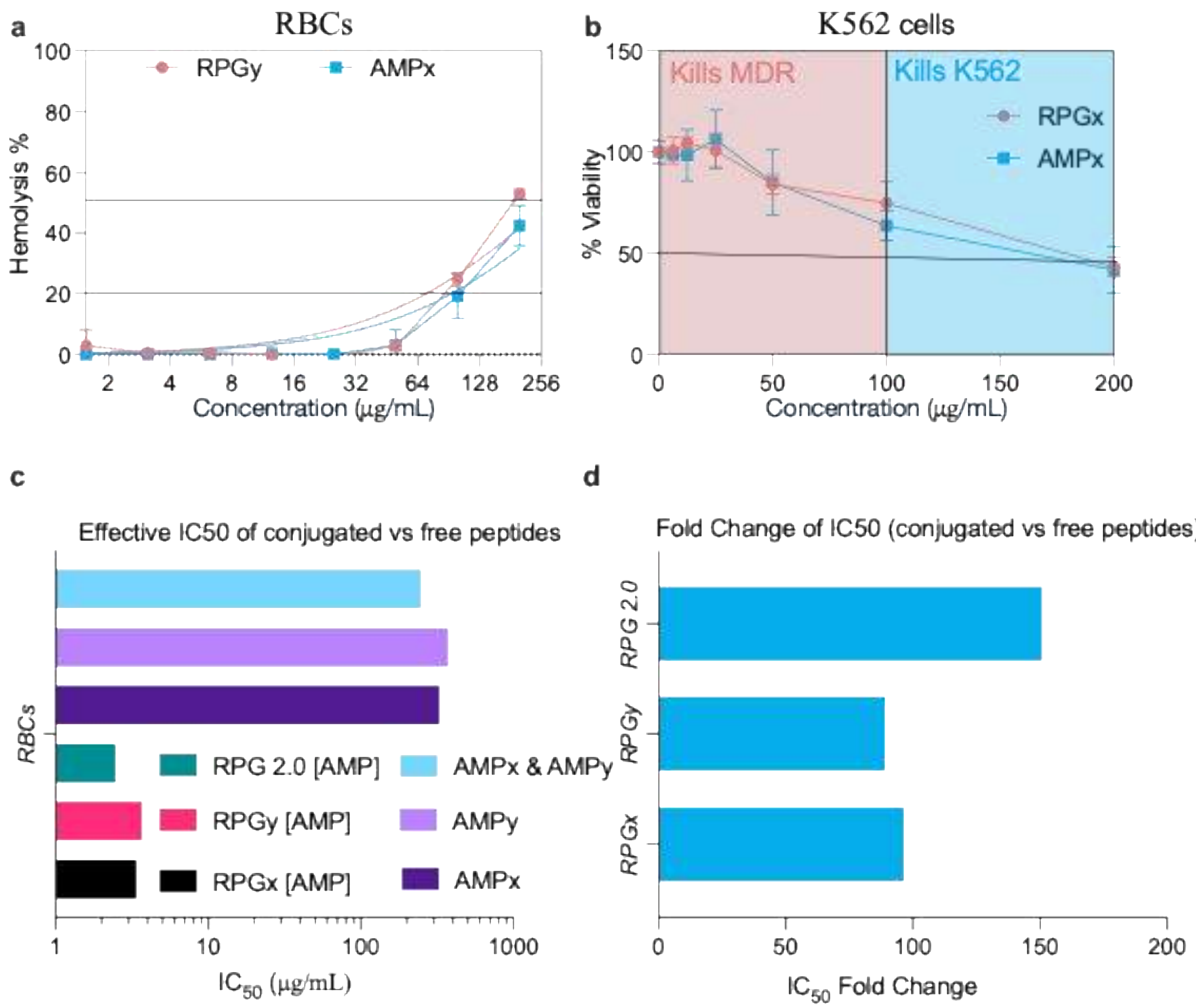

Figure S6 | Cytotoxicity of RPGx and RPGy on mammalian cell lines. a, Hemolytic activity of different concentrations of RPGy or AMPy on red blood cells (RBCs). **b** Cell viability assay testing different concentrations of RPGx or AMPx on K562 suspension cells. **c,** Half-maximal inhibitory concentration (IC_{50}) values for free AMPs and multivalent conjugates (RPG[AMP]) in red blood cells (RBCs). Conjugation to the PVX scaffold increases apparent cytotoxicity on a per-AMP basis. **d,** Fold change of IC_{50} of conjugated AMPx & AMPy relative to free AMPs. Fold change was calculated by dividing the IC_{50} of free AMPx and/or AMPy by the IC_{50} of the absolute

concentration conjugated AMPx and/or AMPy (AMPx and/or AMPy present on the viral surface). All experiments are repeated three times independently with n = 3. RPG, Rod-based Peptide Grids; RBCs, red blood cells.

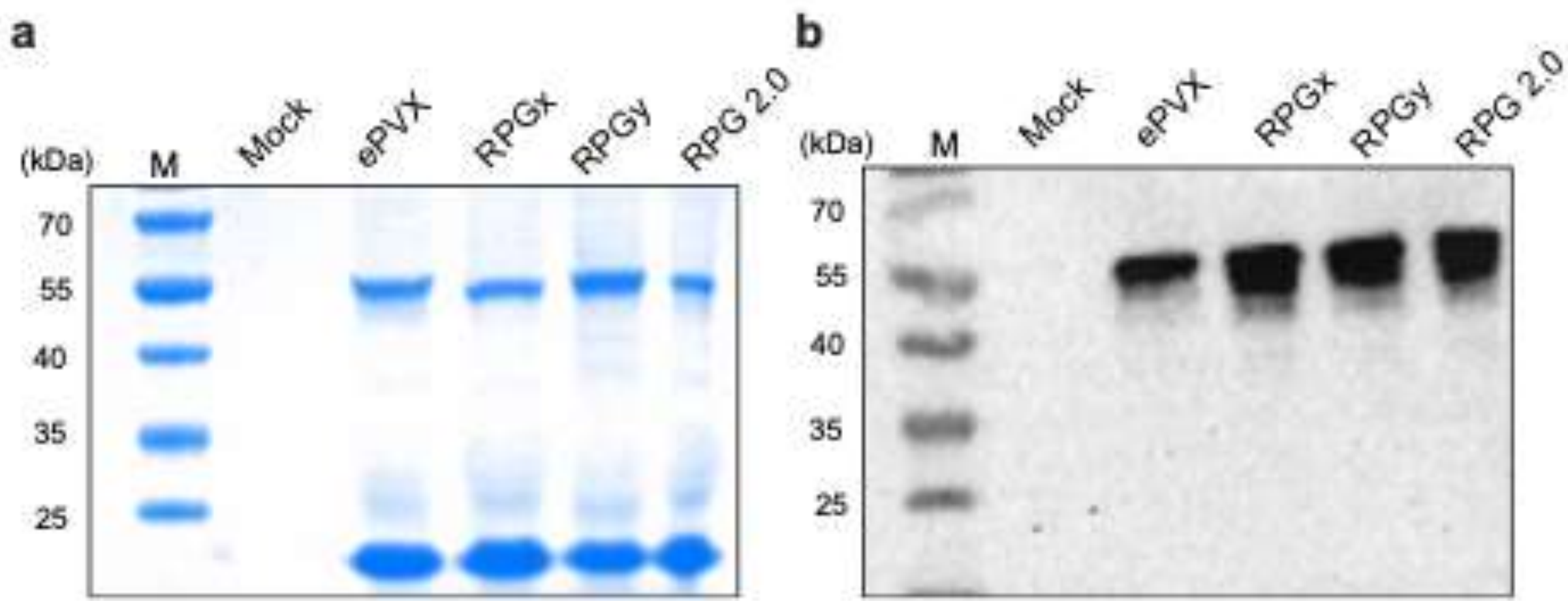

Figure S7 | Gel confirmation of RPG 2.0. a, PAGE gel confirmation of ePVX, RPGx, RPGy, and RPGy. **b**, Westernblot confirmation of ePVX, RPGx, RPGy, and RPGy with AntSpycatcher. RPG, Rod-based Peptide Grids; MIC, minimum inhibitory concentration; MDR, multidrug-resistant.

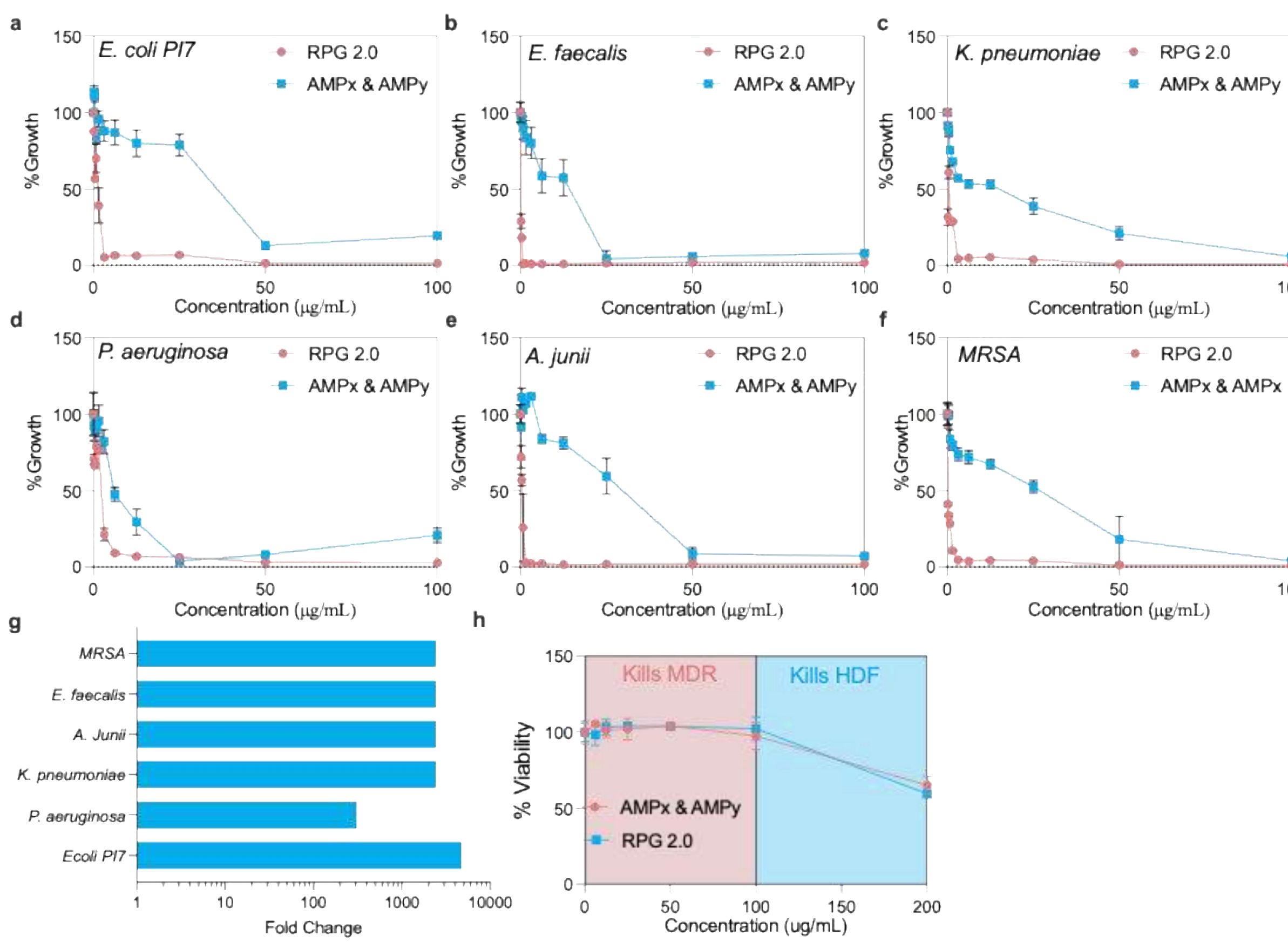

Figure S8 | MIC of RPG 2.0. a-f, MIC of RPG2.0 vs combined free AMPx and Free AMPy on **(a)** *E.coli PI7*, **(b)** *P. aeruginosa*, **(c)** *K. pneumoniae*, **(d)** *A. Junii*, **(e)** *E. faecalis*, and **(f)** *MRSA*. **f**, fold change of the MIC value for conjugated AMP vs free AMPs calculated by dividing the MIC of free AMP over the effective MIC of conjugated AMP (Absolute amount of AMPs on ePVX)—see Fig 4 for the effective MIC of conjugated AMP. **h,** Cell viability assay testing different concentrations of RPG2.0 or AMPx & AMPy on HDF cell line**.** All experiments are repeated three times independently with n = 3. RPG, Rod-based Peptide Grids; MIC, minimum inhibitory concentration; MDR, multidrug-resistant.

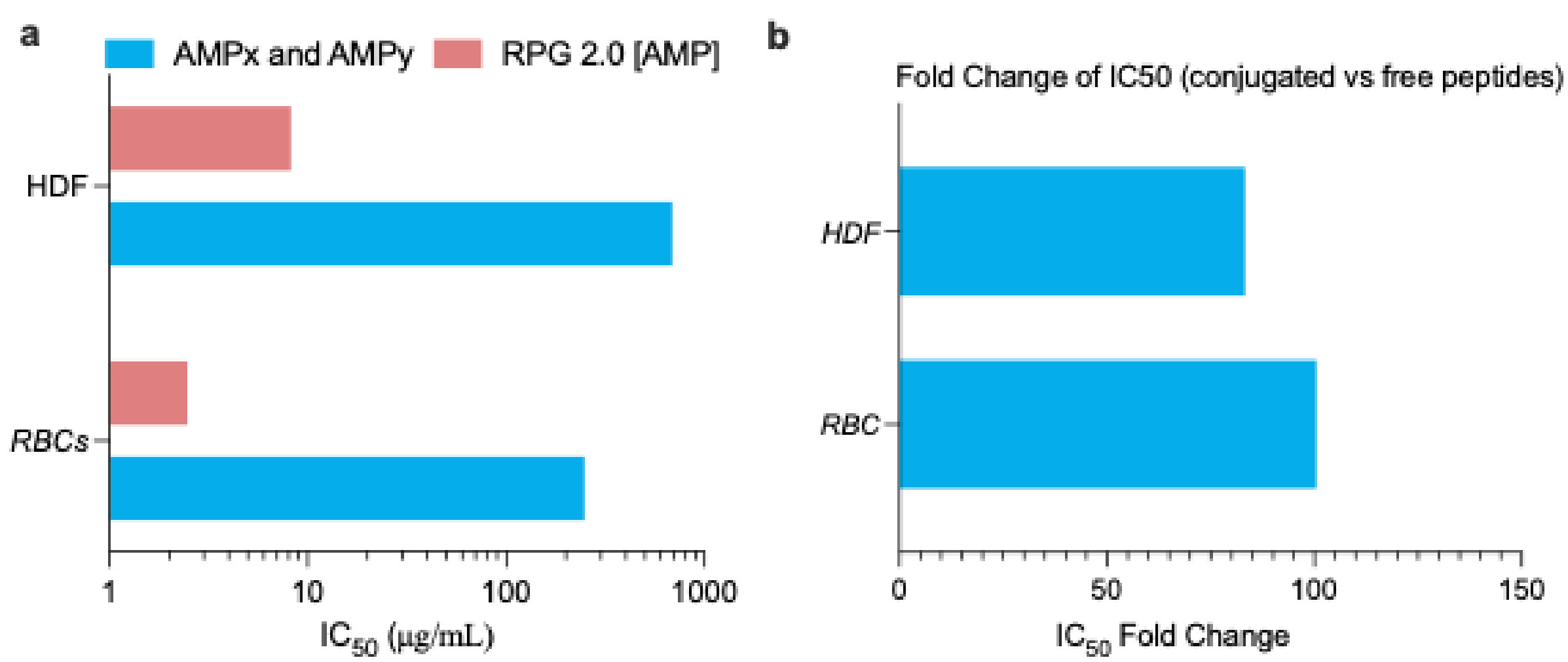

Figure S9 | Therapeutic window and cytotoxicity profile of RPG (conjugated AMPs) relative to free AMPs. a, Half-maximal inhibitory concentration (IC_{50}) values for free AMPs (AMPx & AMPy, blue) and multivalent conjugates (RPG 2.0 [AMP], red) in human dermal fibroblasts (HDF) and red blood cells (RBCs). Conjugation to the PVX scaffold increases apparent cytotoxicity on a per-AMP basis. **b,** Fold change of IC_{50} of conjugated AMPx & AMPy relative to free AMPs. Fold change was calculated by dividing the IC_{50} of free AMPx & AMPy by the IC_{50} of the absolute concentration conjugated AMPx & AMPy (AMPx & AMPy present on the viral surface).

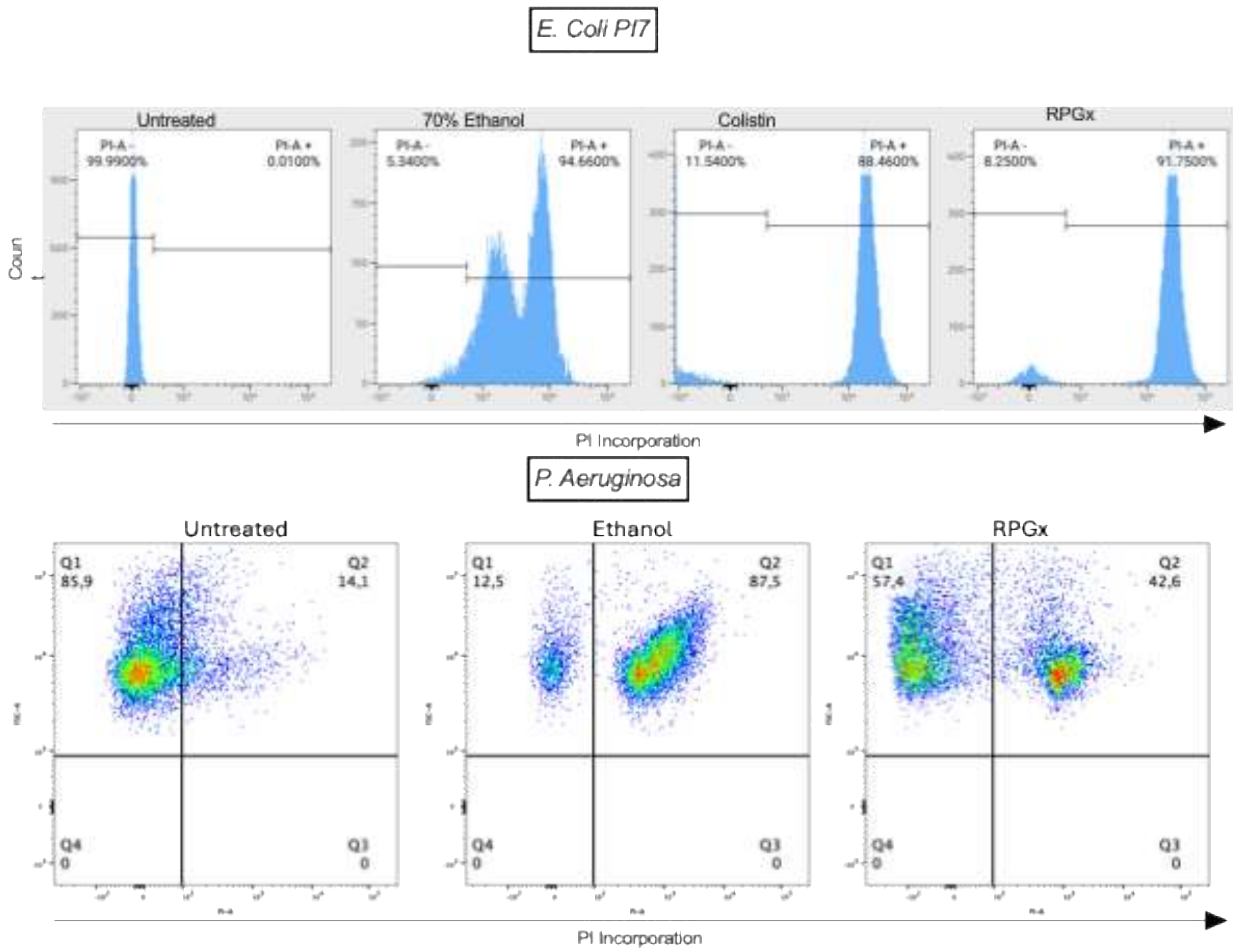

Figure S10 | FACS data of RPGx on *E. coli PI7* and *P. Aeruginosa*. Quantifying the penetration rate of propidium iodide (indicator of cell membrane disruption). Top panel represents FACS data obtained after treating E. coli PI7 with RPGx for 1 hour followed by incubation with propidium iodide. Bottom panel represents FACS data obtained after treating P. aerogo with RPGx for 1 hour followed by incubation with propidium iodide. Untreated samples are bacteria untreated with RPGx or antibiotic. 70% ethanol is used as a positive control as it is known to disrupt membrane. RPG, Rod-based Peptide Grids; FACS, fluorescence-activated cell sorting; PI, propidium iodide.

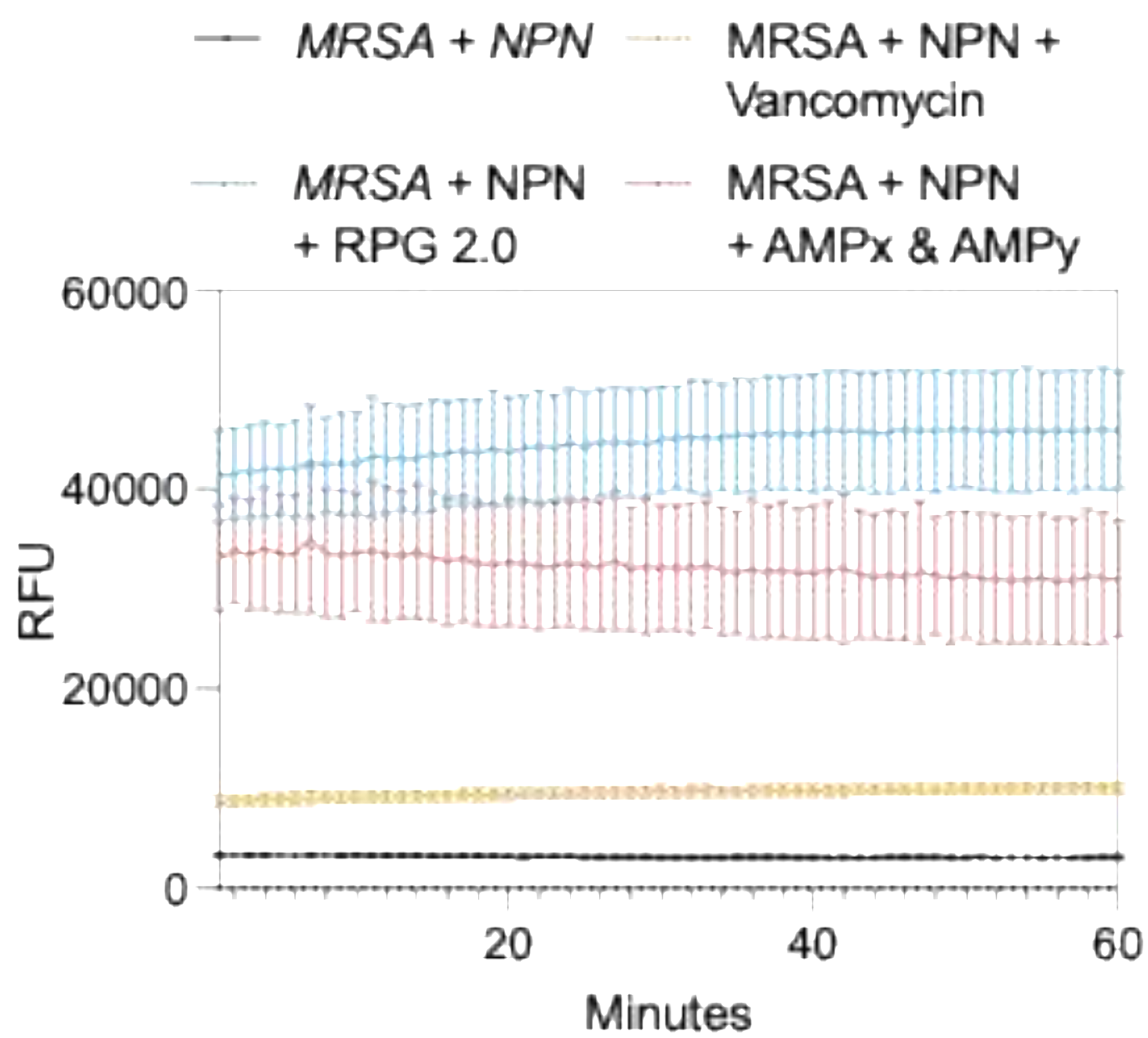

Figure S11 | RPG 2.0 permeabilizes the outer bacterial membrane. NPN fluorescence uptake assay measuring outer membrane permeabilization of *S. aureus* USA300 treated with RPG 2.0 at 2x MIC, Vancomycin at 2x MIC, or left untreated. NPN fluorescence intensity (λ_{ex} = 340 nm, λ_{em} = 405 nm) was recorded every minute for 30 minutes. Increased fluorescence indicates NPN partitioning into the hydrophobic core of destabilized membranes. Vancomycin, which inhibits cell wall biosynthesis without direct membrane perturbation, served as a mechanistically distinct negative control. All experiments were repeated three times independently with n = 3.

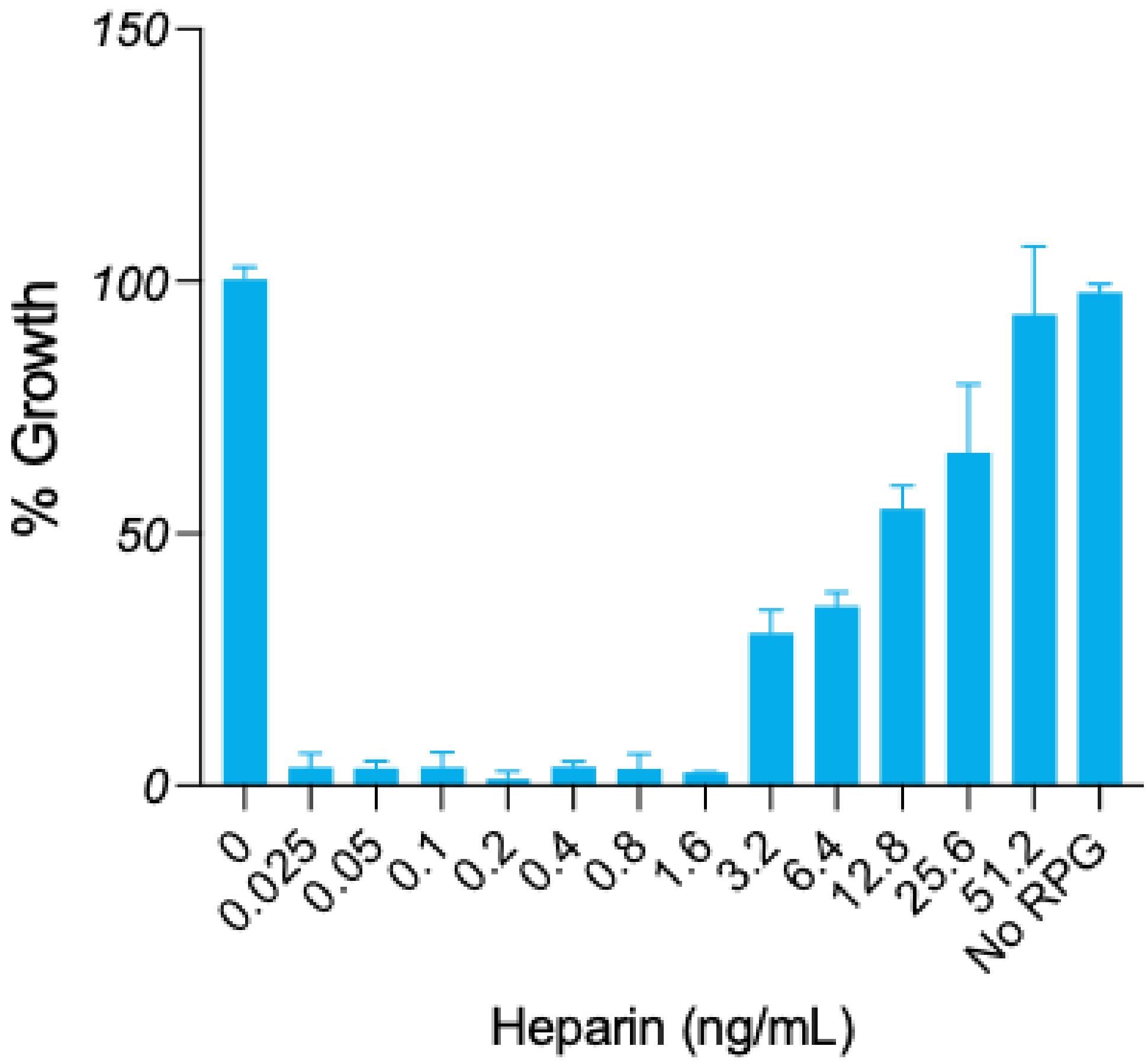

Figure S12 | Charge neutralization of RPG2.0 with heparin. RPG 2.0 was incubated with serial dilutions of a highly sulfated polyanion. Heparin treatment elicited a concentration-dependent attenuation of RPG 2.0 (6.25 µg/mL—2x MIC) activity on *E. coli PI7*. 0 ng/mL is growth control (No RPG, No heparin). No RPG is a heparin toxicity control (Heparin = 51.2 ng/ml). The rest of treatments have RPG 2.0 at 6.25 µg/mL (2x MIC) and serial dilution of heparin in MHB media. The experiment was performed in triplicate with n = 3.

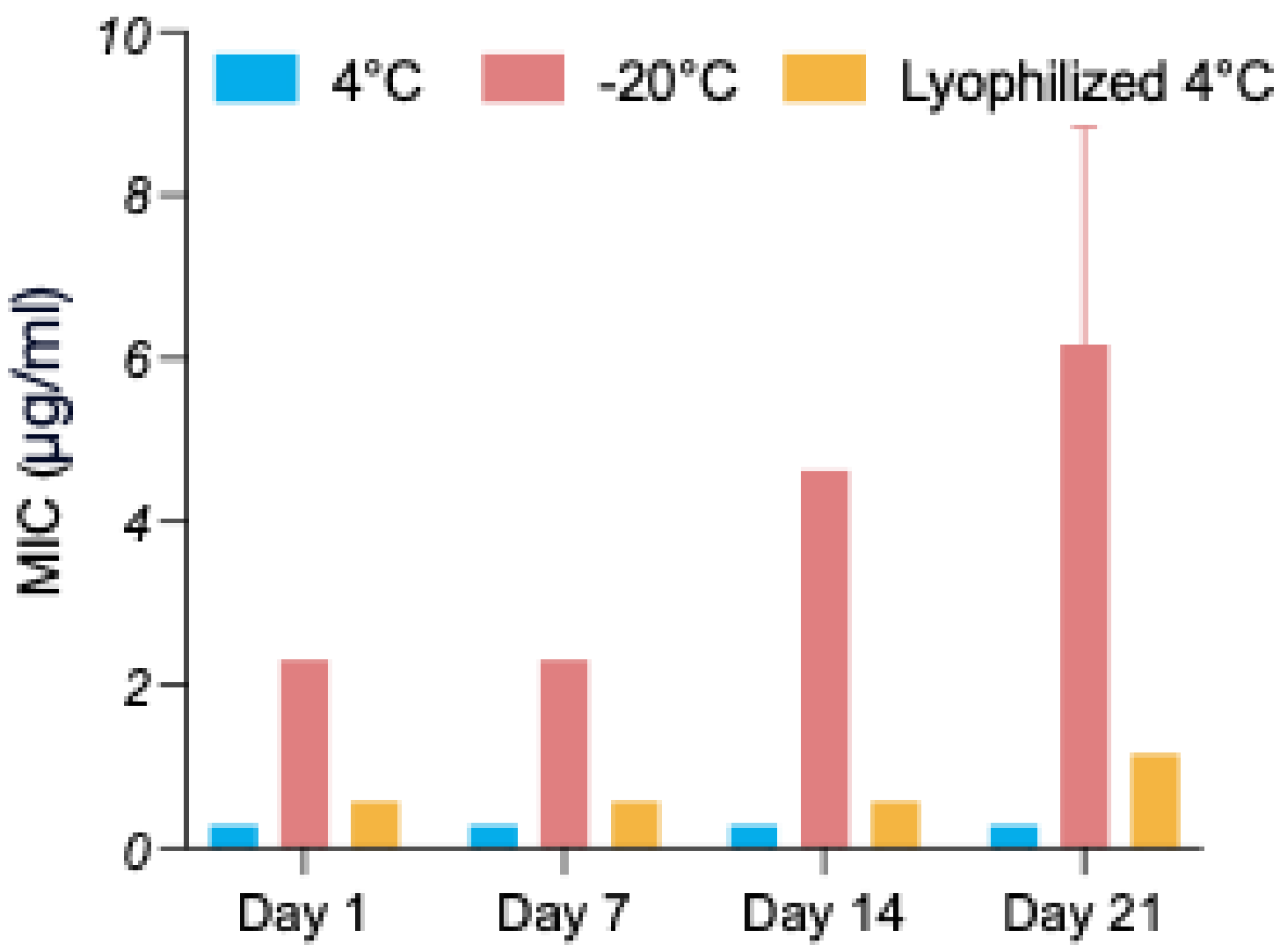

Figure S13 | Long-term storage stability of RPG. Antimicrobial activity of RPG 2.0 stored under three conditions, 4 °C (blue), -20 °C (pink), and lyophilized with 2% trehalose at 4 °C (orange), was monitored over 21 days. Activity is expressed as MIC (µg/mL) against *E. coli PI7*, where lower values indicate retained potency. Data represent mean ± SD from three independent experiments (n = 3).

Table S2. MIC Comparison in µg/mL

Pathogen	RPGy (µg/mL)	Free AMPy (µg/mL)	RPGx (µg/mL)	Free AMPx (µg/mL)	RPG 2.0 (µg/mL)	Free AMPx & AMPy (µg/mL)
MRSA	1.5625	200	100	200	6.25	50
E. faecalis	0.78125	25	100	50	0.78125	25
E. coli PI7	3.215	200	1.5625	200	6.25	200
K. pneumoniae	3.125	200	3.125	200	6.25	100
P. aeruginosa	6.25	200	1.5625	25	25	25
A. junii	1.5625	200	50	100	6.25	50

Table S3. MIC Comparison in Molar Concentration (nM)*

Pathogen	RPGy (nM)	Free AMPy (nM)	RPGx (nM)	Free AMPx (nM)	RPG 2.0 (nM)	Free AMPx & AMPy (nM)
MRSA	0.047	45,662	3	45,662	0.188	11,416
E. faecalis	0.023	5,708	3	11,416	0.023	5,708
E. coli PI7	0.096	45,662	0.047	45,662	0.188	45,662
K. pneumoniae	0.094	45,662	0.094	45,662	0.188	22,831
P. aeruginosa	0.188	45,662	0.047	5,708	0.75	5,708

Pathogen	RPGy (nM)	Free AMPy (nM)	RPGx (nM)	Free AMPx (nM)	RPG 2.0 (nM)	Free AMPx & AMPy (nM)
A. junii	0.047	45,662	1.5	22,831	0.188	11,416

**Molar conversion accounts for the difference in molecular weight (RPG MW ≈ 42,900 kDa; AMPx MW ≈ 4.38 kDa; AMPy MW ≈ 4.38 kDa). RPG is 1270 coat protein (27.33kda each), 444 spycatcher units (14.07kda each) and 444 AMP-spytag unit (4.38 kDa).*

Table S4. MIC Comparison Based on Effective AMP Content (µg/mL AMP equivalents)*

Pathogen	RPGy – Eff. AMP (µg/mL)	Free AMPy (µg/mL)	RPGx – Eff. AMP (µg/mL)	Free AMPx (µg/mL)	RPG 2.0 – Eff. AMP (µg/mL)	Free AMPs (µg/mL)
MRSA	0.0204	200	1.3072	200	0.0817	50
E. faecalis	0.0102	25	1.3072	50	0.0102	25
E. coli PI7	0.0420	200	0.0204	200	0.0817	200
K. pneumoniae	0.0408	200	0.0408	200	0.0817	100
P. aeruginosa	0.0817	200	0.0204	25	0.3268	25
A. junii	0.0204	200	0.6536	100	0.0817	50

**Effective AMP concentration reflects the mass of AMP contained within the RPG dose, calculated from AMP loading (~1.31% by mass). This metric directly compares the antimicrobial potency on a per-peptide basis.*

DNA constructs used in this study

Name	Description	Link
PVX-SpyCatcher	This construct was transformed into Agrobacterium and used to infilterate tobacco plants to produce the ePVX (PVX-SpyCatcher) plattform	https://benchling.com/s/seq-DaNl0db17RKO56yS3gvp?m=slm-n3QYnzVUIRHC2zGoC7aI
wtPVX	This construct was transformed into Agrobacterium and used to infilterate tobacco plants to produce wild type PVX	https://benchling.com/s/seq-93F7o8dU0KwBSrHzSrtS?m=slm-icLU6912FW0izVD1LzMN
SUMO-AMPx-Spytag ecoli	We transformed this construct into E. coli Rosetta cells to produce AMPx	https://benchling.com/s/seq-lak70VdtnS6JMNQgveTI?m=slm-08F7J7TByuksHlWO8L4j
SUMO-AMPy-Spytag Ecoli	We transformed this construct into E. coli Rosetta cells to produce AMPy	https://benchling.com/s/seq-zkzzDm39rHZhE49KMuwu?m=slm-PUfCr56IQrgBWdwHyZfz
SUMO-AMPz-Spytag Ecoli	We transformed this construct into E. coli Rosetta cells to produce AMPz	https://benchling.com/s/seq-kbEUZz2iXRAqhGeEuru3?m=slm-bnGGEABRUggM4EvIQBQg

References

1 de la Fuente-Núñez, C., Reffuveille, F., Haney, E. F., Straus, S. K. & Hancock, R. E. W. Broad-Spectrum Anti-biofilm Peptide That Targets a Cellular Stress Response. *Plos Pathog* **10**, doi:ARTN e1004152
10.1371/journal.ppat.1004152 (2014).

2 Wieczorek, M. *et al.* Structural studies of a peptide with immune modulating and direct antimicrobial activity. *Chem Biol* **17**, 970-980, doi:10.1016/j.chembiol.2010.07.007 (2010).

3 Mansour, S. C., de la Fuente-Nunez, C. & Hancock, R. E. Peptide IDR-1018: modulating the immune system and targeting bacterial biofilms to treat antibiotic-resistant bacterial infections. *J Pept Sci* **21**, 323-329, doi:10.1002/psc.2708 (2015).

4 Chaudhary, S. *et al.* Efficient production of amidated antimicrobial peptides that are active against drug-resistant ESKAPE pathogens. *Nature Communications* **14**, doi:ARTN 1464
10.1038/s41467-023-37003-z (2023).

5 MUNDIM, H. M. Peptideos antimicrobianos IDR 1002 e TS-MAP1: Ressonância Magnética Nuclear para determinação das estruturas tridimensionais. *Tese (Doutorado em Química) - Instituto de Química, Universidade Federal de Goiás, Goiânia*.

6 Turner-Brannen, E. *et al.* Modulation of interleukin-1beta-induced inflammatory responses by a synthetic cationic innate defence regulator peptide, IDR-1002, in synovial fibroblasts. *Arthritis Res Ther* **13**, R129, doi:10.1186/ar3440 (2011).

7 Haney, E. F. *et al.* Computer-aided Discovery of Peptides that Specifically Attack Bacterial Biofilms. *Sci Rep-Uk* **8**, doi:ARTN 1871
10.1038/s41598-018-19669-4 (2018).
